# Divergent Cardiovascular Adaptations and Gene Regulation in High-Elevation Natives and Recent Colonizers of the Qinghai-Tibetan Plateau

**DOI:** 10.1093/molbev/msaf103

**Published:** 2025-05-09

**Authors:** Huishang She, Graham R Scott, Yun Fang, Qingshuo Zhao, Fanwei Meng, Yanhua Qu

**Affiliations:** Key Laboratory of Animal Biodiversity Conservation and Integrated Pest Management, Institute of Zoology, Chinese Academy of Sciences, Beijing, China; Department of Biology, McMaster University, Hamilton, Ontario, Canada; Key Laboratory of Animal Biodiversity Conservation and Integrated Pest Management, Institute of Zoology, Chinese Academy of Sciences, Beijing, China; Key Laboratory of Animal Biodiversity Conservation and Integrated Pest Management, Institute of Zoology, Chinese Academy of Sciences, Beijing, China; Key Laboratory of Animal Biodiversity Conservation and Integrated Pest Management, Institute of Zoology, Chinese Academy of Sciences, Beijing, China; Key Laboratory of Animal Biodiversity Conservation and Integrated Pest Management, Institute of Zoology, Chinese Academy of Sciences, Beijing, China; College of Life Science, University of Chinese Academy of Sciences, Beijing, China

**Keywords:** cardiovascular phenotypes, long-term adaptation, short-term adaptations, high-elevation, gene regulation

## Abstract

High elevation imposes unrelenting and unavoidable hypoxia on species inhabiting these environments, providing an excellent natural setting for studying convergent or divergent evolution. By integrating measures of phenotypic variation, gene regulation, and functional performance, our study demonstrates that recent colonizers of high-elevation environments exhibit fundamentally different cardiovascular changes compared to long-term natives of these environments. Through the studying of heart morphological phenotypes, we showed that recent colonizers exhibit signs of cardiac hypertrophy, reflected by increased relative heart mass (heart mass/body mass) and cardiomyocyte size compared to their low-elevation relatives. In contrast, native species show no signs of cardiac hypertrophy and instead have 3-fold higher capillary densities than the colonizers, a change that likely enhances tissue oxygen diffusing capacity in the former. Using phylogenetic principal component analysis to quantify multivariate trait divergence, we show that native species are similar in cardiovascular phenotype and underlying gene expression, but differ appreciably from recent colonizers. We further demonstrate, using a functional assay, that differential expression of two genes (*IRS2* and *AKT1*) in a conserved regulatory pathway mediates cardiomyocyte hypertrophy, which could explain the observed variation in cardiomyocyte size between native species and recent colonizers. This regulatory basis of variation in cardiac phenotype involves the differential expression of genes in a cardiomyocyte hypertrophy pathway that is conserved across birds, humans and other mammals. Collectively, our study highlights that evolutionary history is a critical determinant of cardiovascular variation in high-elevation environments.

## Introduction

Species may respond similarly or differently to a shared environmental challenge. On the one hand, different species may evolve similar adaptations in response to a common environmental pressure, and this repeated evolution has been considered evidence for selection favoring only a few adaptive solutions (Colosimo et al. 2005; Stern 2013; Bailey et al. 2017; Bolnickn et al. 2018). On the other hand, emerging evidence has revealed that adaptive responses are not always similar (Wang et al. 2015; Zhu et al. 2018; Cheng et al. 2021; Qu et al. 2021), which is regarded as evidence that species may select idiosyncratic solutions in response to a common environmental challenge (Beall 2007). The probability of evolving similar adaptive solutions partially depends on evolutionary history (Zhu et al. 2018; Qu et al. 2021) and the duration of species establishment in that environment (Dawson et al. 2020), as the former determines the evolutionary background on which adaptation builds, and the latter determines the time species have to reach the final adaptive strategy (Zhu et al. 2018).

High-elevation environments provide a unique opportunity to evaluate whether species have followed similar or different pathways in their adaptive evolution (Storz 2021). At high elevations, exposure to hypoxia (reduced oxygen level) leads to plastic physiological adjustments over days to months (e.g. acclimatization, developmental plasticity) in the cardiovascular, respiratory, and metabolic systems. Evolutionary adaptation to high elevation over generations can further refine physiological systems, but the evolutionary pathways of adaptation may differ between lineages. For example, human populations living in the high elevation of Tibet and the Andes have evolved different adaptations to high-elevation environments, as indicated by large quantitative differences in physiological traits involved in the oxygen delivery process (Beall 2007). These distinct evolutionary paths are associated with selection on different suites of genes in Tibetans compared to Andeans (Richalet et al. 2024). Similar observations have been found for non-human animals at high elevation, which exhibit a diverse variety of phenotypic, regulatory, and genetic changes (reviewed in Monge and León-Velarde 1991; Kolar and Ostadal 2007; Storz and Scott 2019). Divergence in adaptation strategies could depend on other distinctions between environments, genetics, and lifestyle (Qu et al. 2015; Velotta et al. 2018; Graham and McCracken 2019; Witt and Huerta-Sánchez 2019; West et al. 2021), but the relative roles of species' evolutionary history and time exposed to the high-elevation environment have rarely been comprehensively explored.

To examine this issue, we have studied a group of passerine birds consisting of two species native to the high-elevation Qinghai-Tibetan Plateau (QTP) and one species that has recently colonized the plateau. Sometimes referred to as the “third polar region” (Wingfield et al. 2011), the QTP has an average elevation of 4,500 m above sea level (a.s.l.) with severe cold temperatures and low oxygen levels (i.e. ∼60% of the oxygen pressure at sea level; Deng et al. 2019). Snowfinches (Aves: Passeridae family) are one of the few avian clades that have diversified in situ on the QTP. The White-rumped Snowfinches (*Onychostruthus taczanowskii*; hereafter referred to as WR-snowfinch) and the Red-necked Snowfinch (*Pyrgilauda ruficollis*; hereafter referred as RN-snowfinch) are endemic species that have inhabited the QTP for 8.3 and 5.7 million years, respectively (Qu et al. 2021). Their closest low-elevation relatives are a group of Old World Sparrows (*Passer*, Päckert et al. 2020). Among them, the Eurasian Tree Sparrow, *Passer montanus*, a human commensal bird commonly found in Eurasia, recently colonized the QTP approximately 2,600 years ago following the establishment of permanent agricultural settlement (Qu et al. 2020).

Compared to low-elevation tree sparrows, the high-elevation tree sparrows (hereafter referred to as recent colonizers) and the two snowfinches (hereafter referred to as natives) exhibit larger body mass and heart mass (Li et al. 2011; Sun et al. 2016). However, whether they exhibit variation in other cardiovascular phenotypes is largely unknown, as are the regulatory mechanisms underlying phenotypic variation. It is possible that these three species exhibit similar cardiovascular changes compared to low-elevation tree sparrows because they all evolved from a common lowland ancestor (i.e. same predefined evolutionary background). Alternatively, evolved differences between species could have led to distinct cardiovascular phenotypes, as they differ in how long they have lived at high elevation (i.e. the time available to reach the final adaptive strategy). For example, it has been shown that some domesticated animals introduced to high elevations have evolved different phenotypes from related non-domesticated species that have lived at high elevation for a longer time (Monge andLeón-Velarde 1991). Such differences could reflect distinct evolutionary pathways of adaptation or different time points along the same adaptive path.

**Fig. 1.**
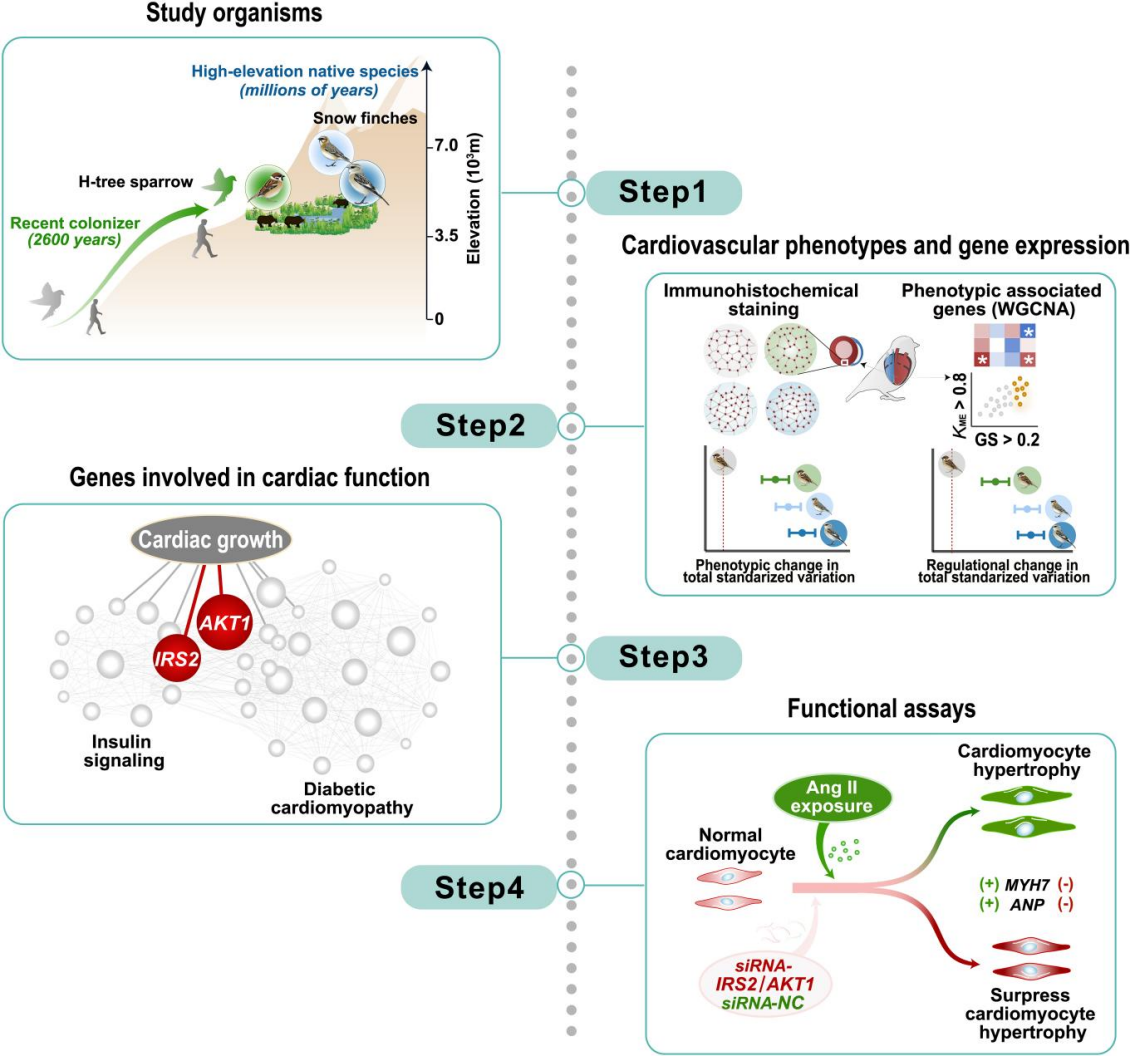
Comparative framework for the integrative analysis of cardiovascular phenotypes. Cardiovascular adaptation in two native species and a recent colonizer of the high-elevation QTP (Step 1) was examined using an integrative approach combining phenotypic data and gene expression analysis (Step 2), network analysis (Step 3), and functional assays (Step 4). The two native species, the White-rumped snowfinch (*Onychostruthus taczanowskii*, WR-snowfinch, dark blue) and Red-necked snowfinch (*Pyrgilauda ruficollis*, RN-snowfinch, light blue), belong to a lineage of snowfinches that have resided on the QTP for millions of years. In contrast, the low-elevation tree sparrow (*Passer montanus*, L-tree sparrow, gray) colonized the QTP much more recently, following the establishment of permanent agricultural settlements approximately 2,600 years ago (H-tree sparrow, green). Using this framework, we found that the two native species exhibit similar cardiovascular phenotypes and gene expression changes, which are highly distinct from those observed in the recent colonizer. A siRNA functional assay demonstrated that differential expression of key genes (*AKT1* and *IRS2*) in a conserved regulatory pathway mediating cardiomyocyte hypertrophy underlies the major phenotypic differences between natives and colonizers.

To investigate these scenarios, we integrated phenotypic data, gene expression, and functional assay data to study cardiovascular phenotypes and gene expression in the two native species and the recent colonizer in comparison to their lowland relative (i.e. the low-elevation tree sparrow). Our results show that the two native species exhibit similar cardiovascular phenotypes and gene expression changes, which are highly distinct from those in the recent colonizer. Using a small interfering RNA (siRNA) functional assay, we demonstrate that the regulatory basis underlying cardiomyocyte hypertrophy, a major phenotypic difference detected between natives and colonizers, is mediated by differential expression of key genes in a conserved regulatory pathway that controls cardiomyocyte hypertrophy (experimental design is shown in Fig. 1). These observations were then considered within the context of previous findings in a broad number of animals from high-elevation environments (i.e. animals in the Himalayas and Andes, reviewed in Monge and León-Velarde 1991; Witt and Huerta-Sánchez 2019). We concluded that cardiovascular phenotypes at high elevation are contingent upon evolutionary history (e.g. high-elevation endemics or human domestic/commensal animals). Our results also suggest that some regulatory bases of cardiovascular change are conserved across the avian and mammalian lineages.

## Results

### High-Elevation Natives and Recent Colonizer Show Different Cardiovascular Phenotypes

**Fig. 2.**
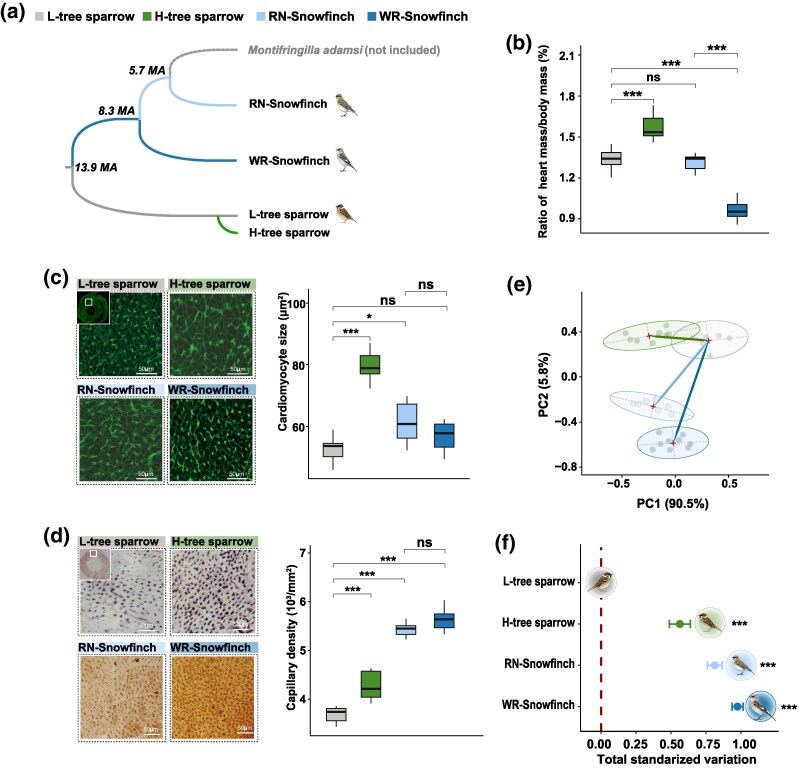
Cardiovascular phenotypes observed in the two native species and a recent colonizer of the QTP. a) The White-rumped snowfinch (WR-snowfinch, dark blue) and Red-necked snowfinch (RN-snowfinch, light blue) are long-duration endemics that have inhabited the QTP for approximately 8.3 and 5.7 million years (Ma), respectively. They belong to a lineage of snowfinches (which also includes the Black-winged snowfinch, *Montifringilla adamsi*). Although *M. adamsi* was not used in this study, it is included here to illustrate the origins of the RN-Snowfinch and WR-Snowfinch. The low-elevation tree sparrow (L-tree sparrow, gray) colonized the QTP following the establishment of permanent human agricultural settlements approximately 2,600 years ago, resulting in the more recently established high-elevation population (H-tree sparrow, green). b) The ratio of heart mass to body mass increased only in the recent colonizer, with no significant change observed in the native species. c) and d) Left panels show representative immunohistochemical images of cardiomyocytes c) and histological images of cardiac capillaries d). Right panels illustrate cardiomyocyte size (cross-sectional area) increased in the recent colonizer compared to L-tree sparrows, while the two native species showed either not change or a slight decrease c). Capillary density increased more in the native species than in the recent colonizer when compared to the L-tree sparrow d). e) Phylogenetic PCA was used to summarize multivariate trait variation and quantify cardiovascular phenotypic divergence. f) The total phenotypic variations of the three high-elevation taxa were calculated relative to the low-elevation tree sparrow (i.e. an approximate ancestral state). For ease of interpretation, 2D morphospaces are plotted in e), but multidimensional morphospaces (including five principal components) were used to calculate total standardized variation in f). b) to d) Statistical significance was assessed using Student's *t*-test withFDR correction: ns, nonsignificance; *P < 0.05; ***P < 0.001. f) Post hoc HSD tests: ns, nonsignificance; ***P < 0.001.

We first examined the ratio of heart mass to body mass (i.e. heart mass/body mass, a measure of cardiac hypertrophy) of the two native species (WR-snowfinch, *n* = 7; RN-snowfinch, *n* = 9), the recent colonizer (*n* = 8), and low-elevation tree sparrows (*n* = 9, Fig. 2a and supplementary table S1, Supplementary Material online). We found that the heart mass/body mass ratio increased in the high-elevation tree sparrow (Student's *t*-test with false discovery rate [FDR] correction, 1.57 vs. 1.34, *P* < 0.001) but decreased in the WR-snowfinch (0.96 vs. 1.34, *P* < 0.001) or remained unchanged in the RN-snowfinch (1.31 vs. 1.34, *P* = 0.58, Fig. 2b and supplementary table S2, Supplementary Material online) compared to the low-elevation tree sparrow. These results suggest that the relative increase in heart mass occurs only in the recent colonizer but not in the two native species.

We then measured and compared cardiomyocyte size (cross-sectional area) in the left ventricles of the two native species and the recent colonizer with that of low-elevation tree sparrows using immunohistochemistry (see Materials and Methods). We found that cardiomyocyte size was significantly increased by 50% in the recent colonizer compared to low-elevation tree sparrows (79.63 vs. 52.64 µm^2^, *P* < 0.001, Fig. 2c and supplementary table S3, Supplementary Material online). In the two native species, we observed no or only a slight increase in cardiomyocyte size compared to low-elevation tree sparrows (WR-snowfinch, 56.49 vs. 52.64 µm^2^, *P* > 0.05; RN-snowfinch, 61.34 vs. 52.64 µm^2^, *P* < 0.05, Fig. 2c and supplementary table S3, Supplementary Material online). Given that cardiomyocyte size correlated with heart mass (Pearson correlation coefficient, *r* = 0.85-0.93, *P* < 0.05, supplementary fig. S1, Supplementary Material online), we ran a linear regression model setting cardiomyocyte size against heart mass and found that the residuals of cardiomyocyte size (after controlling for heart mass) increased significantly in the recent colonizer (0.334 vs. −0.037, *P* < 0.001) but did not change in the RN-snowfinch (−0.068 vs. −0.037, *P* = 0.6) or even decreased in the WR-snowfinch (−0.22 vs. −0.037, *P* < 0.001, supplementary fig. S2, Supplementary Material online). Together, these results indicate that the recent colonizers, but not the native species, exhibited an increase in cardiac mass and cardiomyocyte size, likely due to cardiac hypertrophy.

We then measured cardiac capillary density, a phenotype critical for supporting tissue oxygen transport and aerobic metabolism (Scott et al. 2015; Parr et al. 2019). We observed an increase in capillary density in the left ventricle in the two native species and the recent colonizer compared to the low-elevation tree sparrows (Student's *t*-test and FDR correction, all comparisons, *P* < 0.001, Fig. 2d and supplementary table S4, Supplementary Material online). This increase was more pronounced in the native species than in the recent colonizer, with an approximately 50% increase in the former (5,429 capillaries per mm^2^ in RN-snowfinch and 5,619 capillaries per mm^2^ in WR-snowfinch) but only a 16% increase in the latter (4,280 capillaries per mm^2^ in high-elevation tree sparrows vs. 3,864 capillaries per mm^2^ in low-elevation tree sparrows, supplementary table S4, Supplementary Material online). As capillary density correlated with cardiomyocyte size (Pearson correlation coefficient, *r* = 0.85 to 0.93, *P* < 0.05, supplementary fig. S3, Supplementary Material online), we used linear regression to control for the confounding effect of cardiomyocyte size on capillary density. After controlling for cardiomyocyte size, capillary density was still increased in the native species and recent colonizer, with a much greater in the former than in the latter (supplementary fig. S4, Supplementary Material online). Interestingly, the two native species did not differ from each other in capillary density either before or after controlling for the effect of cardiomyocyte size (*P* > 0.05, Fig. 2d and supplementary table S4, Supplementary Material online). These results suggest that the native species and recent colonizer exhibit different cardiovascular responses to high elevation, either by changing the direction (i.e. presence/absence of cardiac hypertrophy) or the magnitude of phenotypic changes (i.e. different extents of increase in capillary density).

### Cardiovascular Phenotypes are Similar Between Natives but Different from the Recent Colonizer

To quantify phenotypic divergence in a multivariate trait space, we used phylogenetic principal component analysis (PCA) to summarize the five above-mentioned phenotypic measures while accounting for phylogenetic dependence among species (Revell 2009). We constructed a phylogenetic tree using RAxML (Stamatakis 2014) and then used it to calculate phylogenetic PCA scores for visualizing phenotypic variation (i.e. morphospace) across species (Fig. 2e and supplementary fig. S5, Supplementary Material online). Using the low-elevation tree sparrows as the approximate ancestral state, we quantified the change in total standardized variation in morphospaces for each of the three high-elevation taxa relative to the low-elevation tree sparrows (Fig. 2f). We found that the morphospaces occupied by the two native species were more similar to each other (0.81 and 0.97 total standardized variation) and deviated greatly from that occupied by the recent colonizer (i.e. 0.56, post hoc Turkey's honest significant difference [HSD] tests, *P* < 0.001, Fig. 2f). Additionally, the difference between the two native species was smaller than the difference between high-elevation and low-elevation tree sparrows (i.e. 0.15 vs. 0.56, HSD test, *P* < 0.001, Fig. 2f). Together, these results suggest that the changes in cardiovascular phenotype in the two native species are similar but differ appreciably from those in the recent colonizer.

### Transcriptomic Regulation Associated with Changes in Cardiovascular Phenotype

**Fig. 3.**
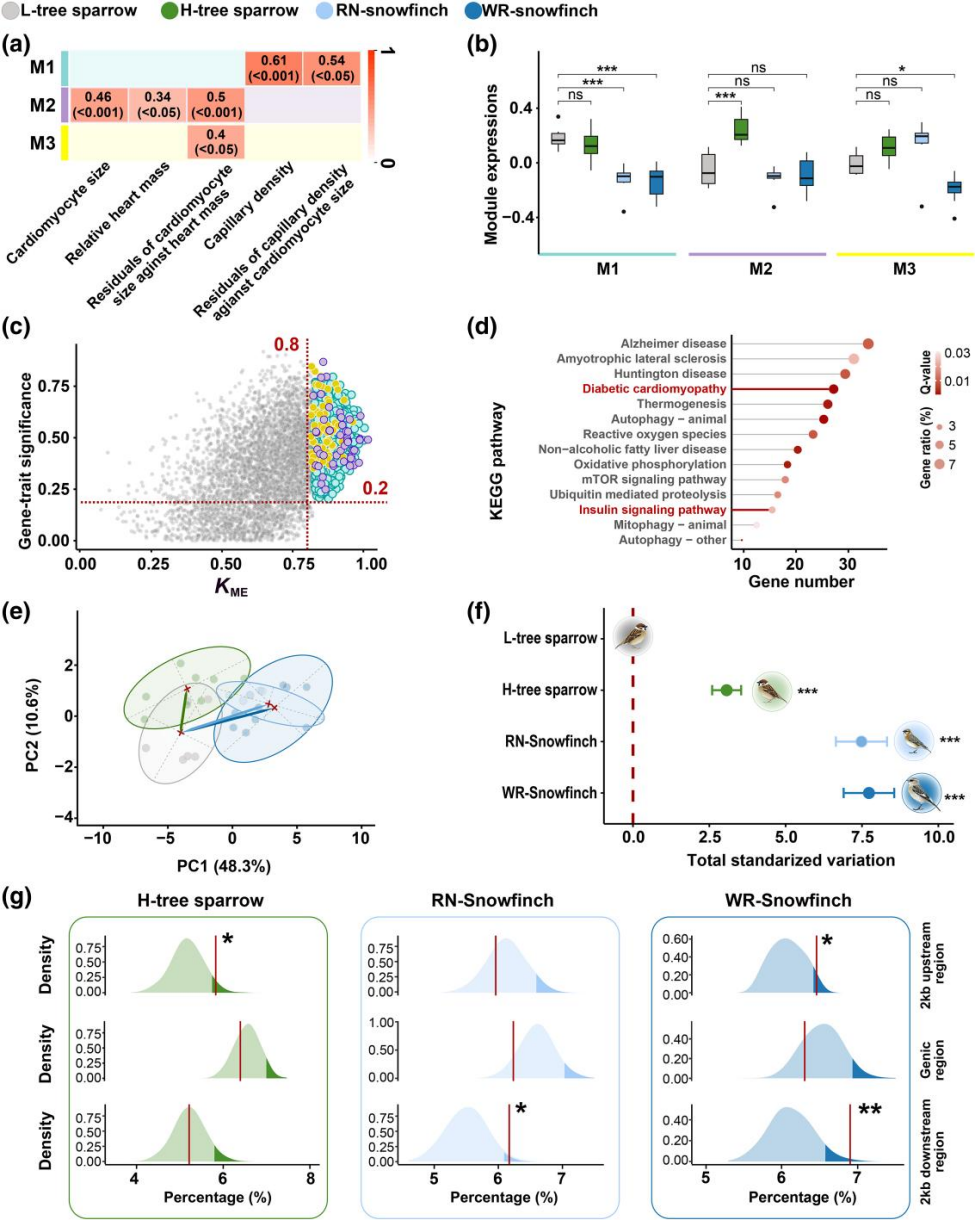
Transcriptomic variation associated with differences in cardiovascular phenotype. a) Three modules of co-expressed genes were identified, with expression levels significantly associated with the observed cardiovascular phenotypes (*R*^2^ ≥ 0.34, *P* < 0.05). b) Overall module expression was significantly associated with the observed cardiovascular phenotypes. Statistical significance was assessed using Student's *t*-test with FDR correction: ns, nonsignificant; *P < 0.05; ***P < 0.001. c) A total of 948 hub genes were identified based on gene significance (GS > 0.2) and module membership (*K*_ME_ > 0.8) for the three modules. The color of each hub gene corresponds to its module color, as indicated in a). d) The hub genes are significantly enriched for functions related to cardiac function, signaling, oxygen metabolism and autophagy. Two pathways involved in cardiac hypertrophy are highlighted in red. e) Phylogenetic PCA was used to summarize gene expression variation. f) The total gene expression variation of the three high-elevation taxa was calculated relative to the low-elevation tree sparrow (total standardized variation). For ease of interpretation, two-dimensional gene expression spaces are plotted in e), but multidimensional gene expression spaces (including 29 principal components) were used to calculate total standardized variation in f). Statistical significance was assessed using post hoc HSD tests: ***P < 0.001. g) Proportions of the selected regions (i.e. genomic regions with top 1% CLR scores) located within 2 kb upstream and/or downstream of the hub genes (vertical red lines) were significantly greater than expected values based on the genomic background in the three high-elevation taxa. This is reflected by proportions exceeding the 95th percentile of the null distributions (*, > 95th percentile of the null distribution, indicated by the dark shaded region of each null distribution; **, > 99th percentile of the null distribution).

We sequenced cardiac transcriptomes to explore the regulatory changes underlying the observed phenotypic changes. We first normalized gene expression across species using scaling factors calculated based on 809 conserved genes (WR-snowfinch vs. tree sparrow, 1.298; RN-snowfinch vs. tree sparrow, 1.374), as described in Hao et al. (2019). Given that variation in cardiovascular phenotypes could be driven by complex coordinated transcriptional responses involving the co-regulation of large modules of genes, we used weighted gene correlation network analysis (WGCNA; Langfelder and Horvath 2008) to analyze the expression data across all species. WGCNA is a systems biology method for identifying groups of highly co-expressed genes (modules), summarizing module-level expression, and identifying important genes within modules (hub genes) that capture expression-phenotype correlations. We identified three modules of co-expressed genes whose expression was significantly associated with at least one cardiovascular phenotype after controlling for species as a random factor (Fig. 3a and supplementary table S5, Supplementary Material online). Module 1 was associated with capillary density and the residuals of capillary density against cardiomyocyte size (*R*^2^ ≥ 0.54, *P* < 0.05) and was expressed at low levels in the two high-elevation native species compared to both high-elevation and low-elevation tree sparrows (Fig. 3b and supplementary fig. S6, Supplementary Material online). Module 2 was associated with the heart mass to body mass, cardiomyocyte size, and the residuals of cardiomyocyte size against heart mass (*R*^2^ ≥ 0.34, *P* < 0.05), and its expression was increased in high-elevation tree sparrows compared to low-elevation tree sparrows and the two species of snowfinches (Fig. 3b and supplementary fig. S6, Supplementary Material online). Module 3 was associated with the residuals of cardiomyocyte size against heart mass (*R*^2^ = 0.4, *P* < 0.05). The overall expression of this module decreased in WR-snowfinch, but remained unchanged in RN-snowfinch, high-elevation and low-elevation tree sparrows (Fig. 3b and supplementary fig. S6, Supplementary Material online).

Based on gene significance (GS > 0.2) and module membership (*K*_ME_ > 0.8), we identified 948 hub genes from the three modules (Fig. 3c). Functional enrichment analysis showed that these hub genes were significantly enriched in cardiac function (e.g. diabetic cardiomyopathy and insulin signaling pathway), signaling (mTOR signaling pathway), oxygen metabolism (thermogenesis and oxidative phosphorylation), autophagy, and diseases (Fig. 3d).

Because the gene expression levels of these hub genes co-varied strongly with cardiovascular phenotypes, we hypothesized that hub gene expression would exhibit a similar overall divergence pattern as the cardiovascular phenotypes. To test this, we carried out phylogenetic PCA to summarize expression changes of the 948 identified hub genes (Fig. 3e). Using the low-elevation tree sparrows as the approximate ancestral state, we quantified the change in total standardized variation in gene expression spaces for each of the three high-elevation taxa relative to the low-elevation tree sparrows (Fig. 3e). We found that the gene expression spaces occupied by the two native species were similar (7.48 vs. 7.72 total standardized variation), but deviated greatly from that of the recent colonizer (i.e. 3.06, HSD test, *P* < 0.001, Fig. 3f). Moreover, the distance between the gene expression spaces of the two native species was much smaller than that between high-elevation and low-elevation tree sparrows (0.24 vs. 3.06, *P* > 0.001, Fig. 3f). Overall, this variation in hub gene expression supports the observations that some gene expression changes in response to high-elevation environment are similar between the two native species but differ from the recent colonizer.

### Accelerated Selection in the Regulatory Regions of the Hub Genes in the High-Elevation Taxa

To assess whether genetic divergence contributes to expression variation of the hub genes, we searched for genomic signatures of selection in each of the three high-elevation taxa. We used SweeD, a method that detects signatures of selection using the composite likelihood ratio (CLR) statistic (Pavlidis et al. 2013). Specifically, we calculated CLR scores for each of the three high-elevation taxa separately, using genome-wide sequencing data available from previous studies (high-elevation tree sparrow, *n* = 11, Qu et al. 2020; WR-snowfinch, *n* = 11; RN-snowfinch, *n* = 11, She et al. 2022) based on a non-overlapping 2-kb sliding window approach. For each taxon, the windows falling into the top 1% of CLR scores were regarded as the genomic regions with significant signatures of selection. We then evaluated the proportion of the selected regions in each taxon associated with the 948 hub genes (including the genic region as well as the regions 2 kb upstream and downstream, Wittkopp and Kalay 2012). If the hub genes have been a target of selection in a given high-elevation taxon, we would expect the identified hub genes to represent a significantly elevated proportion of the selected genomic regions compared to the genomic background. Consistent with this expectation, we found that the 2-kb upstream and/or downstream regions of the hub genes were overrepresented among the genomic regions displaying signatures of selection in each of the three high-elevation taxa (empirical proportions > 95th percentiles of the null distributions, Fig. 3g). Additionally, these regulatory regions of the hub genes exhibited low nucleotide diversity in all three high-elevation taxa (supplementary fig. S7, Supplementary Material online), and the 2-kb downstream regions of H-tree sparrow and RN-snowfinch also showed low Tajima's *D* values compared to the genomic backgrounds (supplementary fig. S8, Supplementary Material online). Together, these results provide evidence that selection has acted on a group of co-expressed genes in each of the three high-elevation taxa, underpinning the observed cardiovascular variation in the high-elevation taxa (i.e. polygenic adaptation).

### Regulatory Changes of Serine/Threonine Kinase 1 (AKT1) and Insulin Receptor Substrate 2 (IRS2) Underlying Cardiomyocyte Hypertrophy

**Fig. 4.**
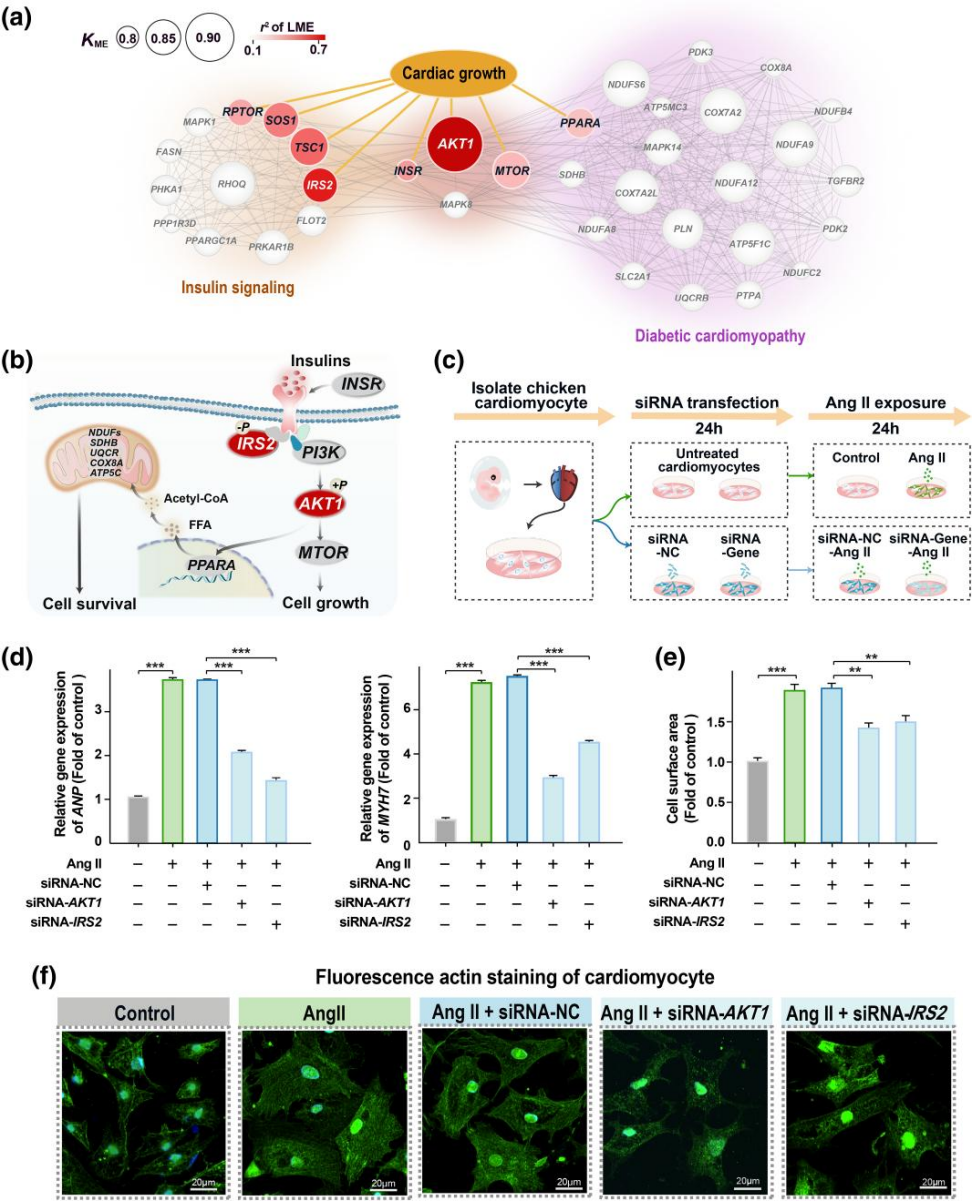
Expression changes of *AKT1* and *IRS2* mediate cardiomyocyte hypertrophy. a) Hub genes in the diabetic cardiomyopathy and insulin signaling pathways. Each circle represents a gene, with its size indicating the module membership (*K*_ME_) of the gene. Eight genes functionally related to cardiac growth are highlighted in red, and the color density represents the correlation coefficient between the residual of cardiomyocyte size against heart mass and the expression level of each gene. b) *AKT1* and *IRS2* are involved in the IR/IGF1R-PI3K-AKT signaling cascade, which regulates developmental and physiological growth of the heart. c) Schematic representation of the *AKT1* and *IRS2* functional experiment using embryonic cardiomyocytes from chicken. We used an in vitro model of cardiomyocyte hypertrophy induced by Ang II. d) to f) After Ang II exposure, siRNA-mediated knockdown of *AKT1* and *IRS2* significantly reduced mRNA levels of gene markers of cardiac hypertrophy d), *ANP* (encoding atrial natriuretic peptide) and *MYH7 (*encoding β-MHC), as well as the surface areas of cardiomyocytes e, f) compared to both Ang II-treated controls and the siRNA negative controls (siRNA-NC). *N* = 3 biological replicates per group. All comparisons were statistically tested using Student's *t*-test with FDR multiple correction: **P < 0.01; ***P < 0.001.

To examine the regulatory basis underlying changes in cardiovascular phenotypes, we focused on genes functionally related to cardiomyocyte hypertrophy, based on our striking observation that cardiomyocyte hypertrophy occurred in the recent colonizer but not in the native species. Of the two pathways related to cardiac function, i.e. diabetic cardiomyopathy and insulin signaling pathways, eight genes were involved in cardiac growth (Fig. 4a and supplementary table S6, Supplementary Material online). These genes exhibited signals of selective sweep in their regulatory or genic regions in one or two of the high-elevation taxa, as indicated by increased CLR values compared to null distributions from 1,000 permutations (empirical values > 95th percentiles of the null distributions generated for each of eight genes, supplementary fig. S9, Supplementary Material online). Two of these genes, *AKT1* and *IRS2*, showed the strongest association between expression and phenotype, and were expressed at lower levels in the native high-elevation species than in the recent high-elevation colonizer (linear mixed model, *AKT1*, *R*^2^ = 0.626, *P* < 0.001; *IRS2*, *R*^2^ = 0.507, *P* < 0.001; supplementary fig. S10 and table S6, Supplementary Material online). *AKT1* exhibited significantly increased CLR values in its regulatory region in H-tree sparrows while *IRS2* showed elevated CLR value in its regulatory region in the WR-snowfinch and RN-snowfinch, compared to their respective null distributions (supplementary fig. S9, Supplementary Material online). These two genes are involved in the IR/IGF1R-PI3K-AKT signaling cascade (Fig. 4b), which regulates developmental and physiological growth of the heart (Condorelli et al. 2002; Boudina et al. 2009; Chen et al. 2010; Nakamura and Sadoshima 2018). Given their important functional roles and the strong association with cardiovascular phenotypes, we selected them for a functional assay to examine whether their regulatory changes mediate avian cardiac hypertrophy.

We established a neonatal chicken cardiomyocyte line and used an in vitro model of cardiomyocyte hypertrophy induced by angiotensin II (Ang II, Fig. 4c), as described in Riehle et al. (2014). To investigate the effect of knockdown of *AKT1* and *IRS2* on Ang II-induced chicken cardiomyocyte hypertrophy, we transfected siRNA-*AKT1* and siRNA-*IRS2* and their siRNA negative controls (siRNA-NC) to the cardiomyocytes. Knockdown efficiency was validated by quantitative polymerase chain reaction (qPCR) (supplementary fig. S11, Supplementary Material online), showing 88.2% and 51.3% reduction in *AKT1* and *IRS2* mRNA levels, respectively, compared to siRNA-NC controls. After exposing these cardiomyocytes to Ang II for 24 h, we observed that Ang II-treated and siRNA-NC cardiomyocytes exhibited increased mRNA levels of gene markers of cardiac hypertrophy: atrial natriuretic peptide (ANP, encoded by *ANP*) and β-myosin heavy chain (β-MHC, encoded by *MYH7*), compared to untreated control cardiomyocytes (Fig. 4d). Furthermore, Ang II treatment also increased the surface areas of the Ang II-treated and siRNA-NC cardiomyocytes compared to untreated control cardiomyocytes (Fig. 4e-f), confirming that Ang II treatment induced cardiomyocyte hypertrophy. In contrast, the knockdown of *AKT1* or *IRS2* suppressed Ang II-induced hypertrophic responses, as indicated by the reduction in ANP and β-MHC mRNA levels and the decrease in surface areas of siRNA-treated cardiomyocytes compared to Ang II-treated and siRNA-NC cardiomyocytes (*n* = 3 biological repeats, Fig. 4d to f). Together, these results suggest that the downregulation of *AKT1* and *IRS2* can counteract Ang II-induced avian cardiomyocyte hypertrophy.

## Discussion

Cardiovascular adaptation is critical for survival in the hypoxic environment of high elevation (Kolar and Ostadal 2007). The tree sparrow, an avian species that recently colonized the QTP, exhibits different cardiovascular responses to the high-elevation environment compared to the two native snowfinch species. For example, the recent colonizer has a large relative heart mass and large cardiomyocyte size compared to low-elevation tree sparrows, a phenotypic change that could enhance the heart's capacity to pump blood (McClelland and Scott 2019). In contrast, the two native species exhibit increased cardiac capillary density with no change in cardiomyocyte size, which could improve oxygen transport and diffusion within the heart (Scott et al. 2011).

Exposure to high-elevation environment could induce phenotypic plasticity, contributing to some of the observed cardiovascular variation. For example, chronic exposure to hypoxia and/or cold has been shown to increase the mass and capillary of the left ventricle in both high-elevation and low-elevation populations of deer mice (Tate et al. 2017; Wearing and Scott 2022). However, evolved changes in high-elevation taxa may have modified these plastic responses to cold and hypoxia, contributing to the observed variation in cardiovascular phenotypes and gene expression (Velotta et al. 2018; Storz and Scott 2019). Indeed, in our previous studies of tree sparrows, we observed that gene expression plasticity induced by hypoxia acclimation in low-elevation tree sparrows can be altered by genetic change in the recent colonizer (Qu et al. 2020; She et al. 2024). In the present study, we also detected signals of selective sweep in the regulatory regions of hub genes in the high-elevation taxa. Although our genetic selection analysis (i.e. SweeD) was limited to a modest sample size (i.e. *n* = 11 for each taxon), we accounted for this by testing for selective sweep based on the distribution across genome-wide sites rather than individual sites, with the former being more robust at smaller sample sizes. The consistent observation of selective sweep pattern across the high-elevation taxa further strengthens the evidence for selection acting on genes related to the variation in cardiovascular phenotypes.

The distinct cardiovascular phenotypes and gene expression observed here may result from the vastly longer duration that snowfinches have inhabited high-elevation environments, providing more time for evolutionary adaptation. Additionally, the variation may reflect different evolutionary histories, as each species has been separated for millions of years. The similarity in cardiovascular phenotypes between the two native species could also be due to their phylogenetic closeness relative to tree sparrows, as the two snowfinch species share a common ancestor likely adapted to high-elevation environment (Päckert et al. 2020; Qu et al. 2021). Thus, the observed cardiovascular phenotypes may reflect the combined effects of plasticity and evolution, potentially influenced by different evolutionary histories and species-specific adaptive trajectories.

The differences in cardiovascular phenotypes distinguishing recent colonizer from high-elevation native species are also observed in other high-elevation animals (Monge and León-Velarde 1991; Kolar and Ostadal 2007). For example, several domestic animals recently introduced to high-elevation environments (e.g. rabbit, lambs, and pigs) show increased relative heart mass compared to their low-elevation counterparts (Hultgren and Miller 1965; Monge and Monge 1968; Anand et al. 1988). This aligns with our observations in tree sparrows, which colonized the QTP alongside the introduction of human agriculture approximately 2,600 years ago (Qu et al. 2020), suggesting that human domestic and commensal animals in high-elevation environments exhibit similar cardiovascular changes (Witt and Huerta-Sánchez 2019). Such changes are absent or less pronounced in long-term high-elevation native species, such as alpaca, llamas, and deer mice (Reynafarje et al. 1968; Banchero et al. 1971; Van Bui and Banchero 1980; Harris et al. 1982; Velotta et al. 2018; West et al. 2021).

The group of high-elevation birds studied here provides a unique opportunity to unravel the regulatory basis for cardiovascular phenotypic variation. Using WGCNA, we identified differentially expressed genes underlying the observed phenotypic changes. We found that the cardiovascular phenotypic changes observed here are mediated by the same regulatory mechanisms underlying cardiac hypertrophy in mice and humans. For example, the IR/IGF1R-PI3K-AKT signaling cascade, a pivotal regulatory hub for cardiac hypertrophy in mice and humans (Heineke and Molkentin 2006), also plays a central role in regulating cardiac hypertrophy in birds. Specifically, using an siRNA functional assay, we demonstrate that two genes in this cascade, *AKT1* and *IRS2*, likely regulate the absence or presence of cardiomyocyte hypertrophy in high-elevation native or colonized birds. These results, together with previous findings that deletion of *IRS2* and *AKT1* causes resistance to exercise- and hypoxia-induced cardiac hypertrophy in mice and rats (Ramasamy et al. 2021), suggest that a conserved regulatory mechanism underlies cardiac hypertrophy in birds and mammals.

In summary, high-elevation native species exhibit distinct cardiovascular responses to high-elevation environments compared to recent colonizers, influenced by their different evolutionary histories and phylogenetic relationship. In the case of cardiomyocyte hypertrophy, phenotypic outcomes are determined by differential expression of key genes in a conserved regulatory pathway. This regulatory basis of cardiovascular variation involves the differential expression of genes in a cardiomyocyte hypertrophy pathway conserved across birds, mice, and humans. Whether other avian and mammalian lineages exhibit similar changes in cardiovascular phenotypes and underlying regulatory mechanisms in high-elevation environment remains to be explored. Further work to elucidate the convergent mechanisms involved in cardiovascular variation across avian and mammalian lineages would provide valuable insights into general mechanisms of environmental adaptation. Such studies may also offer unique perspectives on hypoxia-mediated cardiovascular diseases in humans, particularly considering that birds are highly tolerant of hypoxia to support flight at high elevation (West 2009; Parr et al. 2019).

## Materials and Methods

### Sampling Collection and Phenotypic Comparisons

We collected seven WR-snowfinches, nine RN-snowfinches, and eight high-elevation tree sparrows from Gahai, Qinghai (3,400 m). For low-elevation tree sparrows, we collected six individuals from Beijing (60 m) and three individuals from Wuhu, Anhui (40 m), respectively (supplementary table S1, Supplementary Material online). All these birds were used for phenotypic measures, immunohistological staining, and transcriptomic sequencing. We collected low-elevation tree sparrows from two localities to test whether the difference in cardiovascular phenotypes stemmed from geographic variation rather than high-elevation adaptation. We found no differences between individuals from the two low-elevation localities in any measurement, suggesting no geographic variation in their cardiovascular phenotypes (supplementary fig. S12, Supplementary Material online). Therefore, we combined all low-elevation individuals for subsequent comparative analyses.

### Immunohistological Staining

Hearts were sectioned transversely (10 μm) to epicardium fiber length using a Leica CM1950 cryostat. We used wheat germ agglutinin (WGA, Sigma, USA; 1:100 dilution) staining to visualize cardiomyocytes, as described in Callis et al. (2009). Briefly, slides were fixed with 4% paraformaldehyde for 10 min and then incubated with WGA for 30 min in the dark at room temperature. Cardiomyocytes were stained using wheat germ agglutinin-FITC conjugate, which labeled sarcolemmal membranes with green fluorescence. Cross-sectional area was measured as a quantitative metric of cardiomyocyte size.

Capillaries were identified in the heart using two approaches. In tree sparrows, capillaries were identified by staining using alkaline phosphatase activity (ALP staining: buffer solutions in Mm: 1.0 nitroblue tetrazolium, 0.5 5-bromo-4-chloro-3-indoxyl phosphate, 28 NaBO_2_, 7 MgSO4; pH 9.4) for 1 h at room temperature (Nikel et al. 2018). However, as ALP staining failed for snowfinches, we instead identified capillaries using Griffonia simplicifolia lectins peroxide (GSL-1 staining). Briefly, slides were hydrated with phosphate-buffered saline (PBS; 0.1 M, pH 7.4) and then blocked using Carbo-Free Blocking Solution (Vector Laboratories, Burlingame, CA, USA) at room temperature for 30 min. After staining slides with GSL1 (10 μg/mL in Hepes Buffer with 1 mM GalNAc) for 30 min, they were rinsed with PBS, treated with ABC reagent for 30 min, and subsequently stained with ImmPACT DAN for 2 min. ALP staining and GSL-1 staining have been confirmed to yield statistically equivalent values of capillary density (Nikel et al. 2018).

We measured the cross-sectional areas of cardiomyocytes using a Zeiss LSM710 laser scanning confocal microscope and capillary density using a Leica DM750 light microscope fitted with a Sony camera. The number of images (*n* ≥ 10) was determined by the number needed to yield a stable mean value for each individual.

### Transcriptomic Comparisons

We performed RNA-seq analysis to explore the regulatory basis underlying cardiovascular phenotypic changes in response to high elevation in the two native species and the recent colonizer. Approximately 20 μg RNA was extracted from the left ventricles of all birds. Libraries were constructed according to the manufacturer's protocol (Illumina). As RNA libraries failed for three low-elevation tree sparrows, only the six remaining individuals were included in subsequent analyses (supplementary table S1, Supplementary Material online). RNA sequencing was carried out on seven WR-snowfinches, nine RN-snowfinches, eight high-elevation tree sparrows, and six low-elevation tree sparrows using 150 bp paired-end reads on an Illumina HiSeq 4000 platform. After filtering low-quality, adapter-contaminated, and N-rich reads (*>*10%), cleaned reads were mapped to the genome of each species (Qu et al. 2020, 2021) and gene expression intensity was calculated using Kallisto (Bray et al. 2016). Based on the annotated genomes of WR-snowfinches, RN-snowfinches and tree sparrows (https://www.ncbi.nlm.nih.gov/bioproject/417520), we used the reciprocal best-hit method with an e-value <1e^−10^ and a minimum percentage identity of 30% to identify orthologous genes across the three species. We identified 12,077 orthologous genes. To normalize gene expression across species, we compared transcripts per million across all samples and genes, identifying the most conserved genes as those with a coefficient of variance ≤ 0.3. Based on the 809 conserved genes, we identified scaling factors by adjusting median expression values to a common value (WR-snowfinch vs. tree sparrow, 1.298; RN-snowfinch vs. tree sparrow, 1.374), which were subsequently used to scale gene expression across all samples^31^.

We used WGCNA v. 1.41-1 (Langfelder and Horvath 2008) to identify regulatory modules exhibiting correlations with cardiovascular phenotypes. Specifically, based on the 12,077 orthologous genes, we calculated Pearson correlations between each pair of genes and constructed regulatory networks. We used a soft threshold method and computed an adjacency matrix to construct an approximately scale-free topology. Topological overlap was used to create cluster dendrograms based on hierarchical clustering method. Modules were identified as cluster tree branches using the dynamic tree-cutting method. To identify regulatory modules associated with phenotypic traits, we used a PCA to summarize modules of gene expression using the *blockwiseModules* function in WGCNA. The first principal component (PC1) represented overall module expression; therefore, module eigengene values of PC1 were used to test for associations between module expression and cardiovascular phenotypes using a linear mixed model using R package lme4, with species included as a random factor. Regulatory modules were identified with a *P* < 0.05 and a *R*^2^ > 0.3. To identify hub genes (i.e. genes showing strong association with cardiovascular phenotypes), we calculated module membership (*K*_ME_, an indicator of eigengene-based module connectivity) and GS, considering genes with *K*_ME_ > 0.8 and GS > 0.2 as hub genes, as recommended by WGCNA. Functional enrichment analysis of the hub genes was performed using clusterProfiler (Wu et al. 2021).

For each of the eight genes involved in cardiomyocyte hypertrophy (i.e. diabetic cardiomyopathy and insulin signaling pathways, Fig. 4a), we used linear mixed model to examine the relationship between gene expression and phenotypic changes (residual of cardiomyocyte size against heart mass) in the L-tree sparrow, H-tree sparrows, and two species of snowfinches (supplementary fig. S10, Supplementary Material online).

### Phylogenetic PCA

To quantify multivariate variation in cardiovascular phenotypes and gene expression traits across species, we performed a phylogenetic PCA on (i) the five cardiovascular phenotypes and (ii) the 948 hub genes identified by the WGCNA. Non-phylogenetic PCA assumes that samples consist of independent data points, which is likely violated by phylogenetic data from related species. Phylogenetic PCA incorporates this nonindependence among species by assuming a multivariate Brownian motion process of trait evolution and using the expected covariance among traits to calculate principal component axes and scores (Revell 2009). However, we acknowledge that phylogenetic PCA may not fully remove the influence of phylogenetic relationship given the limited species number (e.g. three species). Thus, similarities in cardiovascular phenotypes and gene expression between the two snowfinches may also stem from their phylogenetic closeness.

To estimate phylogenetic relationship, we extracted single nucleotide polymorphisms from transcriptomic data and constructed a maximum likelihood phylogenetic tree using RAxML v. 8.1 (Stamatakis 2014) with the GTR + GAMMA model and 100 bootstraps (supplementary fig. S5, Supplementary Material online). Based on this phylogenetic tree, we performed phylogenetic PCA and calculated the phylogenetic PCA scores for these species using the R package phytools (Revell 2012).

We used phylogenetic PCA scores to construct phenotypic morphospaces and gene expression spaces occupied by each taxon. To account for variance from sampling size (i.e. six low-elevation tree sparrows and nine RN-snowfinches) and matrices (i.e. five phenotypic traits or 948 gene expression traits), we generated effect size describing changes (mean and standard deviation) in phenotypic morphospaces and gene expression spaces for each taxon using Monte Carlo simulation of P-matrices with function “rnorm” function in R. This permutation produced an effect size from 5,000 simulated values for each taxon.

To quantify how phenotypic and gene expression variation may change in response to high-elevation environment, we used the morphospace or gene expression space of low-elevation tree sparrow as the approximate ancestor from which the high-elevation species originated. We then calculated the change in total standardized variation using all PC axes (morphospace, 5 PCs; gene expression space: 29 PCs) between that of low-elevation tree sparrow and that of each of the two native species and the recent colonizer. We used HSD tests to test for significant differences.

### Detecting Signatures of Selection in Hub Genes

For sequence analyses, we used genome-wide sequencing data available for these species from Qu et al. (2020) and She et al. (2022) (high-elevation tree sparrows, *n* = 11; WR-snowfinch, *n* = 11; RN-snowfinch, *n* = 11). Resequencing data for each high-elevation taxon were mapped to its respective genome, and variants were called with GATK HaplotypeCaller for each high-elevation taxon, as described in Qu et al. (2020) and She et al. (2022). We used SweeD (Pavlidis et al. 2013), a method based on the site frequency spectrum, to detect signature of selection for each of the three high-elevation taxa. We calculated CLR values for each high-elevation taxon using a 2-kb sliding window. We applied a threshold based on the empirical distribution of the top 1% of CLR values to detect genomic regions with signatures of selection. We then evaluated the proportions of the highly selective genomic regions associated with the 948 hub genes (including the genic regions as well as the 2-kb upstream and downstream regions of the genic regions) for each of the three high-elevation taxa. These upstream and downstream regions were included because genetic variation in these nearby genic regions can affect gene regulation (Wittkopp and Kalay 2012).

To test for statistical enrichment of CLR outliers within genic and regulatory regions of hub genes, we generated a null distribution of expected proportion for each of the three high-elevation taxa using a randomization resampling procedure. We randomly resampled the same number of genomic windows and calculated the proportions of these randomly sampled regions within the genic, 2-kb upstream or downstream regions of hub genes. This procedure was repeated 1,000 times and generated a null distribution for each high-elevation taxon. The empirical proportion of each high-elevation taxon was compared to its permuted null distribution and proportion greater than 95th percentile of the null distribution was considered statistically significant.

For each of the eight genes involved in cardiac growth (Fig. 4a), we calculated CLR values for regulatory (2-kb upstream and downstream region) and genic regions for each of the three high-elevation taxa. To test statistical significance, we generated null distributions for the regulatory and genic regions of all genes by randomly sampling the same number of windows 1,000 times and permuted CLR distributions. Our permutations were constrained to the genic regions, 2-kb upstream and downstream regions of all genes. Empirical CLR values were compared to permuted CLR distributions generated from the 1,000 random samplings and a threshold of *P* < 0.05 was considered to be statistically significant (empirical CLR > 95th percentile of permutated CLR distributions). All analyses were conducted using the R statistical software package.

Additionally, we calculated nucleotide diversity (π) and Tajima's *D* values (in a 2-kb window size) for the downstream, upstream, and genic regions of the hub genes using VCFtools (Danecek et al. 2011). For comparison, we also calculated these values for the regulatory and genic regions of all genes in the genome, which served as the genomic background. We then compared the nucleotide diversity and Tajima's *D* values of the regulatory and genic regions between the hub genes and the genomic backgrounds for each of the three high-elevation taxa. To determine whether the regulatory or genic regions of the hub genes exhibited significantly lower nucleotide diversity and Tajima's *D* values compared to the genomic background, we performed a Wilcoxon Rank-Sum test.

### SiRNA Functional Assay

We isolated cardiomyocytes from 10-d-old chicken embryos following a protocol described in Liu et al. (2014). Cardiomyocytes were transfected with 80 nmol/L siRNA-*AKT1*, siRNA-*IRS2*, or the negative control siRNA-NC (non-targeting scrambled siRNA designed to have no homology to any known gene sequences) using RNAimax Transfection Reagent (Invitrogen, USA) according to the manufacturer's instructions. Transfection efficiency was evaluated using qPCR (supplementary fig. S11, Supplementary Material online).

We subsequently exposed the siRNA-*AKT1*, siRNA-*IRS2*, siRNA-NC, and normal cardiomyocytes to angiotensin II (Ang II; 500 nmol/L, DRVYIHPF, China) for 24 h (Hu et al. 2019; Gao et al. 2020), while untreated cardiomyocytes served as additional controls to show baseline and hypertrophy induction, respectively. After Ang II treatment, we measured the surface areas of the cardiomyocytes using F-actin staining. Briefly, cardiomyocytes were fixed in 4% formaldehyde in PBS and incubated with 0.05% Triton X-100 for 20 min. They were then incubated with anti-actinin primary antibody (1:500, Proteintech, USA) at 4 °C for 1 h, and visualized using laser confocal microscopy (Andor Dragonfly 505). Chicken embryos were obtained from Boehringer Ingelheim (Beijing, China). siRNA oligonucleotides specific for *AKT1* and *IRS2* were synthesized by OBIO (Shanghai, China). The siRNA sequences used are listed in supplementary table S7, Supplementary Material online. Each experiment was independently repeated three times, with technical triplicates for qPCR and cardiomyocyte surface area measurements. Data were presented as mean and standard errors.

### Real-Time PCR

Total RNA was extracted from cardiomyocytes using TRIzol reagent (Invitrogen, USA), and RNA integrity was assessed using a Nanodrop spectrophotometer. cDNA was synthesized by using PrimeScript RT Master Mix (Takara, Japan). mRNA levels of *AKT1*, *IRS2*,*ANP* and β *MYH7* were quantified using SYBR Green PCR Master Mix (Roche), with glyceraldehyde-3-phosphate dehydrogenase mRNA levels used as a reference. Quantitative real-time PCR was performed using the CFX96 Real-Time PCR detection system (Bio-Rad, USA) to determine the levels of genes of interest, calculated using the comparative quantification method as described in Hu et al. (2019). The primer sequences used in RT-PCR are listed in supplementary table S8, Supplementary Material online.

## Supplementary Material

msaf103_Supplementary_Data

## Data Availability

RNA sequencing data used in this study have been deposited in Short Read Archive under the project number PRJNA417520 (https://www.ncbi.nlm.nih.gov/bioproject/PRJNA417520/). The codes used in this study can be found at https://github.com/shelfey/Cardiovascular-Adaptation-in-Highland-birds/blob/main/cardiac_adaptation.R.

## References

[msaf103-B1] Anand I., Heath D, Williams D, Deen M, Ferrari R, Bergel D, Harris P. The pulmonary circulation of some domestic animals at high altitude. Int J Biometeorol. 1988:32(1):56–64. 10.1007/BF01623996. 3391700

[msaf103-B2] Bailey SF, Blanquart F, Bataillon T, Kassen R. What drives parallel evolution? How population size and mutational variation contribute to repeated evolution. Bioessays. 2017:39(1):1–9. 10.1002/bies.201600176. 27859467

[msaf103-B3] Banchero N, Grover RF, Will JA. Oxygen transport in the llama (*Lama glama*). Respir Physiol. 1971:13(1):102–115. 10.1016/0034-5687(71)90067-3. 5112825

[msaf103-B4] Bolnickn DI, Barrett RDH, Oke KB, Rennison DJ, Stuart YE. (Non)parallel evolution. Annu Rev Ecol Evol. 2018:49(1):303–330. 10.1146/annurev-ecolsys-110617-062240.

[msaf103-B5] Beall CM . Two routes to functional adaptation: Tibetan and Andean high-altitude natives. Proc Natl Acad Sci U S A. 2007:104(suppl_1):8655–8660. 10.1073/pnas.0701985104. 17494744 PMC1876443

[msaf103-B6] Boudina S, Bugger H, Sena S, O'Neill BT, Zaha VG, Ilkun O, Wright JJ, Mazumder PK, Palfreyman E, Tidwell TJ, et al Contribution of impaired myocardial insulin signaling to mitochondrial dysfunction and oxidative stress in the heart. Circulation. 2009:119(9):1272–1283. 10.1161/CIRCULATIONAHA.108.792101. 19237663 PMC2739097

[msaf103-B7] Bray NL, Pimentel H, Melsted P, Pachter L. Near-optimal probabilistic RNA-Seq quantification. Nat Biotechnol. 2016:34(5):525–527. 10.1038/nbt.3519. 27043002

[msaf103-B8] Callis TE, Pandya K, Seok HY, Tang R-H, Tatsuguchi M, Huang Z-P, Chen J-F, Deng Z, Gunn B, Shumate J, et al MicroRNA-208a is a regulator of cardiac hypertrophy and conduction in mice. J Clin Invest. 2009:119(9):2772–2786. 10.1172/JCI36154. 19726871 PMC2735902

[msaf103-B9] Chen PC, Wakimoto H, Conner D, Araki T, Yuan T, Roberts A, Seidman CE, Bronson R, Neel BG, Seidman JG, et al Activation of multiple signaling pathways causes developmental defects in mice with a noonan syndrome–associated sos1 mutation. J Clin Invest. 2010:120(12):4353–4365. 10.1172/JCI43910. 21041952 PMC2993597

[msaf103-B10] Cheng YL, Miller MJ, Zhang D, Xiong Y, Hao Y, Jia C, Cai T, Li S-H, Johansson US, Liu Y, et al Parallel genomic responses to historical climate change and high elevation in east Asian songbirds. Proc Natl Acad Sci U S A. 2021:118(50):e2023918118. 10.1073/pnas.2023918118. 34873033 PMC8685689

[msaf103-B11] Colosimo PF, Hosemann KE, Balabhadra S, Villarreal G, Dickson M, Grimwood J, Schmutz J, Myers RM, Schluter D, Kingsley DM. Widespread parallel evolution in sticklebacks by repeated fixation of ectodysplasin alleles. Science. 2005:307(5717):1928–1933. 10.1126/science.1107239. 15790847

[msaf103-B12] Condorelli G, Drusco A, Stassi G, Bellacosa A, Roncarati R, Iaccarino G, Russo MA, Gu Y, Dalton N, Chung C, et al Akt induces enhanced myocardial contractility and cell size in vivo in transgenic mice. Proc Natl Acad Sci U S A. 2002:99(19):12333–12338. 10.1073/pnas.172376399. 12237475 PMC129445

[msaf103-B13] Danecek P, Auton A, Abecasis G, Albers CA, Banks E, DePristo MA, Handsaker RE, Lunter G, Marth GT, Sherry ST, et al The variant call format and VCFtools. Bioinformatics. 2011:27(15):2156–2158. 10.1093/bioinformatics/btr330. 21653522 PMC3137218

[msaf103-B14] Dawson NJ, Alza L, Nandal G, Scott GR, McCracken KG. Convergent changes in muscle metabolism depend on duration of high-altitude ancestry across Andean waterfowl. eLife. 2020:9:e56259. 10.7554/eLife.56259. 32729830 PMC7494360

[msaf103-B15] Deng L, Zhang C, Yuan K, Gao Y, Pan Y, Ge X, He Y, Yuan Y, Lu Y, Zhang X, et al Prioritizing natural-selection signals from deep-sequencing genomic data suggests multi-variant adaptation in Tibetan highlanders. Nat Sci Rev. 2019:6(6):1201–1222. 10.1093/nsr/nwz108. PMC8291452 34691999

[msaf103-B16] Gao XQ, Zhang Y-H, Liu F, Ponnusamy M, Zhao X-M, Zhou L-Y, Zhai M, Liu C-Y, Li X-M, Wang M, et al The piRNA CHAPIR regulates cardiac hypertrophy by controlling METTL3-dependent N6-methyladenosine methylation of Parp10 mRNA. Nat Cell Biol. 2020:22(11):1319–1331. 10.1038/s41556-020-0576-y. 33020597

[msaf103-B17] Graham AM, McCracken KG. Convergent evolution on the hypoxia-inducible factor (HIF) pathway genes EGLN1 and EPAS1 in high-altitude ducks. Heredity (Edinb). 2019:122(6):819–832. 10.1038/s41437-018-0173-z. 30631144 PMC6781116

[msaf103-B18] Hao Y, Xiong Y, Cheng Y, Song G, Jia C, Qu Y, Lei F. Comparative transcriptomics of 3 high-altitude passerine birds and their low-altitude relatives. Proc Natl Acad Sci U S A. 2019:116(24):11851–11856. 10.1073/pnas.1819657116. 31127049 PMC6576129

[msaf103-B19] Harris P, Heath D, Smith P, Williams DR, Ramirez A, Krüger H, Jones DM. Pulmonary circulation of the llama at high and low altitudes. Thorax. 1982:37(1):38–45. 10.1136/thx.37.1.38. 7071792 PMC459242

[msaf103-B20] Heineke J, Molkentin JD. Regulation of cardiac hypertrophy by intracellular signaling pathways. Nat. Rev. 2006:7(8):589–600. 10.1038/nrm1983. 16936699

[msaf103-B21] Hu S, Cheng M, Guo X, Wang S, Liu B, Jiang H, Huang C, Wu G. Down-regulation of miR-200c attenuates AngII-induced cardiac hypertrophy via targeting the MLCK-mediated pathway. J Cell Mol Med. 2019:23(4):2505–2516. 10.1111/jcmm.14135. 30680929 PMC6433679

[msaf103-B22] Hultgren HN, Miller H. Right ventricular hypertrophy at high altitude. Ann N Y Acad Sci. 1965:127(1):627–631. 10.1111/j.1749-6632.1965.tb49428.x. 4222026

[msaf103-B23] Langfelder P, Horvath S. WGCNA: an R package for weighted correlation network analysis. BMC Bioinf. 2008:9(1):559. 10.1186/1471-2105-9-559. PMC2631488 19114008

[msaf103-B24] Li D, Wu J, Zhang X, Ma X, Wingfield JC, Lei F, Wang G, Wu Y. Comparison of adrenocortical responses to acute stress in lowland and highland Eurasian tree sparrows (*Passer montanus*): similar patterns during the breeding, but different during the prebasic molt. J Exp Zool. 2011:315A(9):512–519. 10.1002/jez.699. 21815272

[msaf103-B25] Liu C, Xue R, Wu D, Wu L, Chen C, Tan W, Chen Y, Dong Y. REDD1 attenuates cardiac hypertrophy via enhancing autophagy. Biochem Biophys Res Commun. 2014:454(1):215–220. 10.1016/j.bbrc.2014.10.079. 25450383

[msaf103-B26] Kolar F, Ostadal B. Cardiac adaptation to chronic high-altitude hypoxia: beneficial and adverse effects. Respir Physiol Neurobiol. 2007:158(2-3):224–236. 10.1016/j.resp.2007.03.005. 17442631

[msaf103-B27] McClelland GB, Scott GR. Evolved mechanisms of aerobic performance and hypoxia resistance in high-altitude natives. Annu Rev Physiol. 2019:81(1):561–583. 10.1146/annurev-physiol-021317-121527. 30256727

[msaf103-B28] Monge CM, León-Velarde F. Physiological adaptation to high altitude: oxygen transport in mamamls and birds. Physiol Rev. 1991:71(4):1135–1172. 10.1152/physrev.1991.71.4.1135. 1924550

[msaf103-B29] Monge C Sr., Monge C Jr. Adaptation to high altitude. In: Hafez ESE, editor. Adaptation of domestic animals. Philadelphia: Lea and Febiger Press; 1968. p. 194–201.

[msaf103-B30] Nakamura M, Sadoshima J. Mechanisms of physiological and pathological cardiac hypertrophy. Nat Rev Cardiol. 2018:15(7):387–407. 10.1038/s41569-018-0007-y. 29674714

[msaf103-B31] Nikel KE, Shanishchara NK, Ivy CM, Dawson NJ, Scott GR. Effects of hypoxia at different life stages on locomotory muscle phenotype in deer mice native to high altitudes. Comp Biochem Physiol B Biochem Mol Biol. 2018:224:98–104. 10.1016/j.cbpb.2017.11.009. 29175484

[msaf103-B32] Päckert M, Favre A, Schnitzler J, Martens J, Sun Y-H, Tietze DT, Hailer F, Michalak I, Strutzenberger P. “Into and out of” the Qinghai-Tibet Plateau and the Himalayas: centers of origin and diversification across five clades of Eurasian montane and alpine passerine birds. Ecol Evol. 2020:10(17):9283–9300. 10.1002/ece3.6615. 32953061 PMC7487248

[msaf103-B33] Parr N, Wilkes M, Hawkes LA. Natural climbers: insights from avian physiology at high altitude. High Alt Med Biol. 2019:20(4):427–437. 10.1089/ham.2019.0032. 31618107

[msaf103-B34] Pavlidis P, Živkovic D, Stamatakis A, Alachiotis N. Sweed: likelihood-based detection of selective sweeps in thousands of genomes. Mol Biol Evol. 2013:30(9):2224–2234. 10.1093/molbev/mst112. 23777627 PMC3748355

[msaf103-B35] Qu Y, Chen C, Xiong Y, She H, Zhang YE, Cheng Y, DuBay S, Li D, Ericson PGP, Hao Y, et al Rapid phenotypic evolution with shallow genomic differentiation during early stages of high elevation adaptation in Eurasian Tree Sparrows. Natl Sci Rev. 2020:7(1):113–127. 10.1093/nsr/nwz138. 34692022 PMC8289047

[msaf103-B36] Qu Y, Chen C, Chen X, Hao Y, She H, Wang M, Ericson PGP, Lin H, Cai T, Song G, et al The evolution of ancestral and species-specific adaptation in snowfinches at the Qinghai-Tibetan Plateau. Proc Natl Acad Sci U S A. 2021:118(13):e2012398118. 10.1073/pnas.2012398118. 33753478 PMC8020664

[msaf103-B37] Qu Y, Tian S, Han N, Zhao H, Gao B, Fu J, Cheng Y, Song G, Ericson PGP, Zhang YE, et al Genetic responses to seasonal variation in altitudinal stress: whole**-**genome resequencing of great tit in eastern Himalayas. Sci Rep. 2015:5(1):14256. 10.1038/srep14256. 26404527 PMC4585896

[msaf103-B38] Ramasamy S, Ramasamy KS, Veerappan M, Chidambaranathan GP. Egr-1 mediated cardiac miR-99 family expression diverges physiological hypertrophy from pathological hypertrophy. Exp Cell Res. 2021:405(2):112709. 10.1016/j.yexcr.2021.112709. 29481791

[msaf103-B39] Revell LJ . Size-correction and principal components for interspecific comparative studies. Evolution. 2009:63(12):3258–3268. 10.1111/j.1558-5646.2009.00804.x. 19663993

[msaf103-B40] Revell LJ . Phytools: an R package for phylogenetic comparative biology (and other things). Methods Ecol Evol. 2012:3(2):217–223. 10.1111/j.2041-210X.2011.00169.x.

[msaf103-B41] Reynafarje C, Faura J, Paredes A, Villavicencio D. Erythrokinetics in high-altitude-adapted animals (llama, alpaca, and vicuna). J Appl Physiol. 1968:24(1):93–97. 10.1152/jappl.1968.24.1.93. 5635776

[msaf103-B42] Richalet JP, Hermand E, Lhuissier FJ. Cardiovascular physiology and pathophysiology at high altitude. Nat Rev Cardiol. 2024:21(2):75–88. 10.1038/s41569-023-00924-9. 37783743

[msaf103-B43] Riehle C, Wende AR, Zhu Y, Oliveira KJ, Pereira RO, Jaishy BP, Bevins J, Valdez S, Noh J, Kim BJ, et al Insulin receptor substrates are essential for the bioenergetic and hypertrophic response of the heart to exercise training. Mol Cell Biol. 2014:34(18):3450–3460. 10.1128/MCB.00426-14. 25002528 PMC4135616

[msaf103-B44] She H, Jiang Z, Song G, Ericson PGP, Luo X, Shao S, Lei F, Qu Y. Quantifying adaptive divergence of the snowfinches in a common landscape. Divers Distrib. 2022:28(12):2579–2592. 10.1111/ddi.13383.

[msaf103-B45] She H, Hao Y, Song G, Luo X, Lei F, Zhai W, Qu Y. Gene expression plasticity followed by genetic change during colonization a high-elevation environment. e-Life. 2024:12:RP86687. 10.7554/eLife.86687. 38470231 PMC10932543

[msaf103-B46] Stern DL . The genetic causes of convergent evolution. Nat Rev Genet. 2013:14(11):751–764. 10.1038/nrg3483. 24105273

[msaf103-B47] Storz JF . High-altitude adaptation: mechanistic insight from integrated genomics and physiology. Mol Biol Evol. 2021:38(7):2677–2691. 10.1093/molbev/msab064. 33751123 PMC8233491

[msaf103-B48] Storz JF, Scott GR. Life ascending: mechanism and process in physiological adaptation to high-altitutde hypoxia. Annu Rev Ecol Evol Syst. 2019:50(1):503–526. 10.1146/annurev-ecolsys-110218-025014. 33033467 PMC7540626

[msaf103-B49] Sun YF, Ren Z-P, Wu Y-F, Lei F-M, Dudley R, Li D-M. Flying high: limits to flight performance by sparrows on the Qinghai-Tibet Plateau. J Exp Biol. 2016:219(Pt 22):3642–3648. 10.1242/jeb.142216. 27609759

[msaf103-B50] Scott GR, Elogio TS, Lui MA, Storz JF, Cheviron ZA. Adaptive modifications of muscle phenotype in high-altitude deer mice are associated with evolved changes in gene regulation. Mol Biol Evol. 2015:32(8):1962–1976. 10.1093/molbev/msv076. 25851956 PMC4592356

[msaf103-B51] Scott GR, Schulte PM, Egginton S, Scott ALM, Richards JG, Milsom WK. Molecular evolution of cytochrome c oxidase underlies high-altitude adaptation in the Bar-headed goose. Mol Biol Evol. 2011:28(1):351–363. 10.1093/molbev/msq205. 20685719

[msaf103-B52] Stamatakis A . RAxML version 8: a tool for phylogenetic analysis and post-analysis of large phylogenies. Bioinformatics. 2014:30(9):1312–1313. 10.1093/bioinformatics/btu033. 24451623 PMC3998144

[msaf103-B53] Tate KB, Ivy CM, Velotta JP, Storz JF, McClelland GB, Cheviron ZA, Scott GR. Circulatory mechanisms underlying adaptive increases in thermogenic capacity in high-altitude deer mice. J Exp Biol. 2017:220(Pt 20):3616–3620. 10.1242/jeb.164491. 28839010 PMC5665433

[msaf103-B54] Van Bui M, Banchero N. Effects of chronic exposure to cold or hypoxia on ventricular eights and ventricular myoglobin concentrations in guinea pigs during growth. Pflugers Arch. 1980:385(2):155–160. 10.1007/BF00588696. 7190274

[msaf103-B55] Velotta JP, Ivy CM, Wolf CJ, Scott GR, Cheviron ZA. Maladaptive phenotypic plasticity in cardiac muscle growth is suppressed in high-altitude deer mice. Evolution. 2018:72(12):2712–2727. 10.1111/evo.13626. 30318588

[msaf103-B56] Wang MS, Li Y, Peng MS. Genomic analyses reveal potential independent adaptation to high altitude in Tibetan chickens. Mol Biol Evol. 2015:32(7):1880–1889. 10.1093/molbev/msv071. 25788450

[msaf103-B57] Wearing OH, Scott GR. Evolved reductions in body temperature and the metabolic costs of thermoregulation in deer mice native to high altitude. Proc Biol Sci. 2022:289(1983):20221553. 10.1098/rspb.2022.1553. 36168757 PMC9515628

[msaf103-B58] West JB . Comparative physiology of the pulmonary blood-gas barrier: the unique avian solution. Am J Physiol Regul Integr Comp Physiol. 2009:297(6):R1625–R1634. 10.1152/ajpregu.00459.2009. 19793953 PMC2803621

[msaf103-B59] West CM, Ivy CM, Husnudinov R, Scott GR. Evolution and developmental plasticity of lung structure in high-altitude deer mice. J Comp Physiol B. 2021:191(2):385–396. 10.1007/s00360-021-01343-3. 33533958

[msaf103-B60] Wingfield JC, Patrick Kelley J, Angelier F, Chastel O, Lei F, Lynn SE., Miner B, Davis JE., Li D, Wang G. Organism-environment interactions in a changing world: a mechanistic approach. J Ornithol. 2011:152(S1):279–288. 10.1007/s10336-011-0668-3.

[msaf103-B61] Witt KE, Huerta-Sánchez E. Convergent evolution in human and domesticate adaptation to high-altitude environments. Philos Trans R Soc Lond B Biol Sci. 2019:374(1777):20180235. 10.1098/rstb.2018.0235. 31154977 PMC6560271

[msaf103-B62] Wittkopp P, Kalay G. Cis-regulatory elements: molecular mechanisms and evolutionary processes underlying divergence. Nat Rev Genet. 2012:13(1):59–69. 10.1038/nrg3095. 22143240

[msaf103-B63] Wu T, Hu E, Xu S, Chen M, Guo P, Dai Z, Feng T, Zhou L, Tang W, Zhan L, et al clusterProfiler 4.0: a universal enrichment tool for interpreting omics data. Innovation. 2021:2:100141. 10.1016/j.xinn.2021.100141. 34557778 PMC8454663

[msaf103-B64] Zhu XJ, Guan Y, Signore AV, Natarajan C, DuBay SG, Cheng Y, Han N, Song G, Qu Y, Moriyama H. Divergent and parallel routes of biochemical adaptation in high-altitude passerine birds from the Qinghai-Tibet Plateau. Proc Natl Acad Sci U S A. 2018:115(8):1865–1870. 10.1073/pnas.1720487115. 29432191 PMC5828625

