# Supplementary material for: Divergent Cardiovascular Adaptations and Gene Regulation in High-Elevation Natives and Recent Colonizers of the Qinghai-Tibetan Plateau
Source: Mol Biol Evol. 2025 May 9;42(5):msaf103. doi: 10.1093/molbev/msaf103 (PMC12103977; doi:10.1093/molbev/msaf103)
Supplement: msaf103_Supplementary_Data [file msaf103_supplementary_data.zip › MBE-2024-1048.R2-cardiac adaptation_Supporting information.pdf]

Supplementary Materials for

Divergent cardiovascular adaptations and gene regulation in high-elevation natives and recent colonizers of the Qinghai-Tibetan Plateau

Huishang She[1], Graham R. Scott[2], Yun Fang[1], Qingshuo Zhao[1], Fanwei Meng[1], Yanhua Qu[1,3]

[1] Key Laboratory of Animal Biodiversity Conservation and Integrated Pest Management, Institute of Zoology, Chinese Academy of Sciences, Beijing, China
[2] Department of Biology, McMaster University, Hamilton, Ontario, Canada
[3] College of Life Science, University of Chinese Academy of Sciences, Beijing, China
Corresponding authors: Fanwei Meng (mengfw@ioz.ac.cn) and Yanhua Qu (quyh@ioz.ac.cn)

Fig. S1

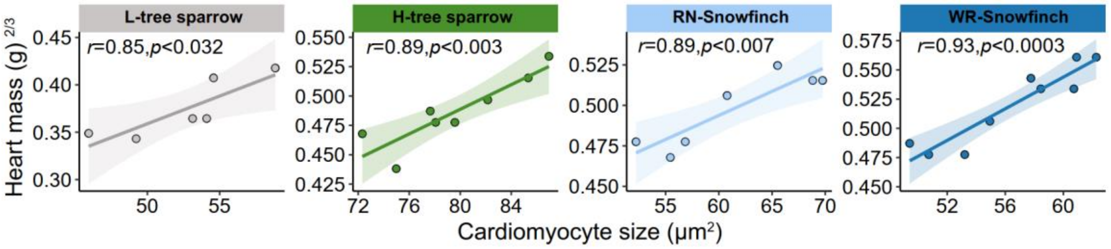

Fig. S1 Cardiomyocyte size (cross-sectional area) correlated significantly with heart mass in the White-rumped Snowfinch (WR-snowfinch), Red-necked Snowfinch (RN-snowfinch) and low-elevation tree sparrows (L-tree sparrow) and high-elevation (H-tree sparrow). Pearson correlation was used to test the correlation between cardiomyocyte size and heart mass to the 2/3 power.

Fig. S2

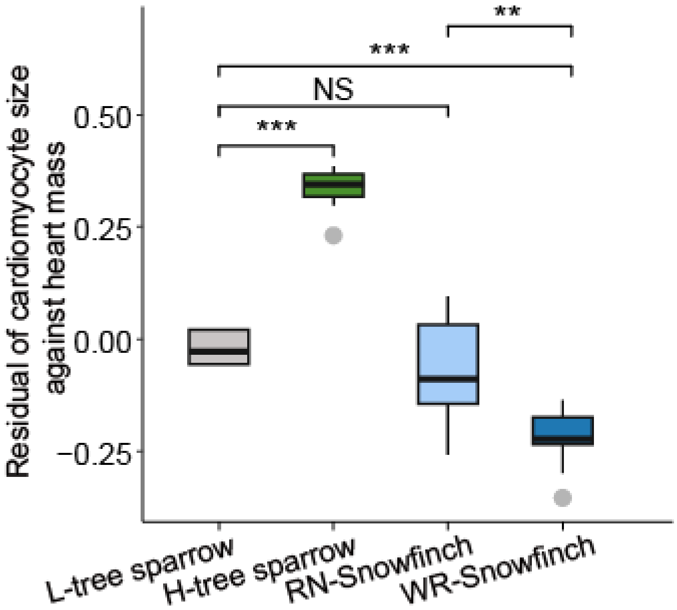

Fig. S2 The residual of cardiomyocyte size after controlling heart mass only significantly increased in the colonizer (H-tree sparrow), but did not change in red-necked snowfinch (RN-snowfinch) or even decreased in white-rumped snowfinch (WR-snowfinch).

Fig. S3

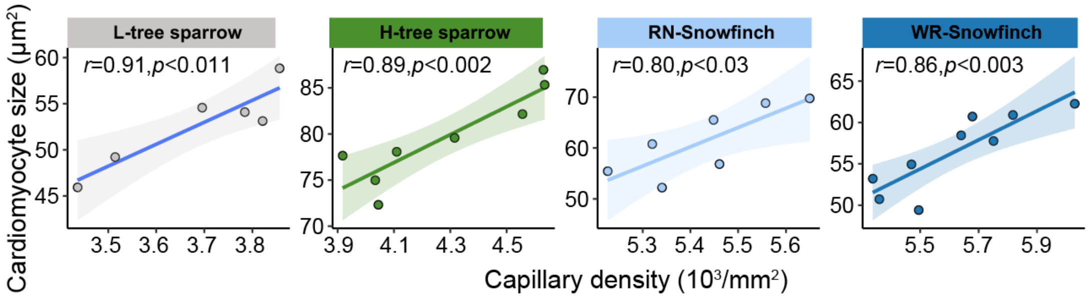

Fig. S3 Capillary density correlated significantly with cardiomyocyte size in the White-rumped snowfinch (WR-snowfinch), Red-necked snowfinch (RN-snowfinch) and low-elevation tree sparrows (L-tree sparrow) and high-elevation tree sparrows (H-tree sparrow). Pearson correlation was used to test the correlation between cardiomyocyte size and capillary density.

Fig. S4

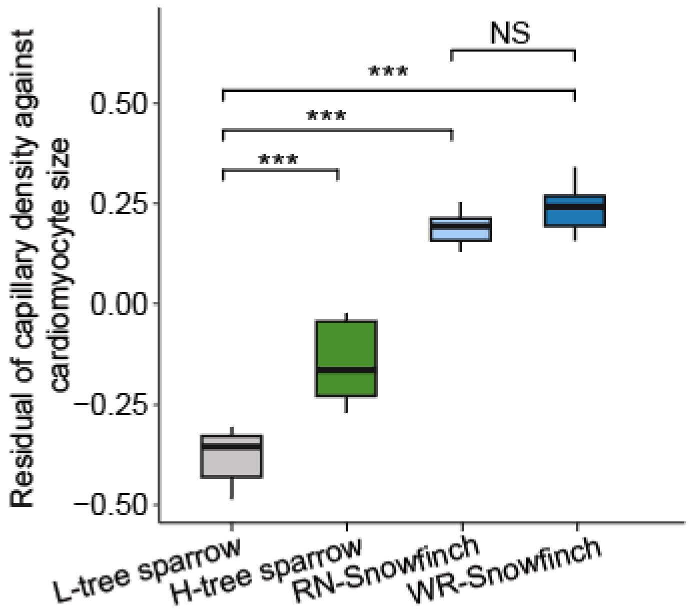

Fig. S4 The residuals of capillary density after controlling for cardiomyocyte size only significantly change between the recent colonizers (H-tree sparrow) and native species (RN-snowfinch and WR-snowfinch), but do not change in the two natives. Statistical significance was assessed using Student's *t*-test with false discovery rate (FDR) correction: ns, nonsignificance; ***, $P<0.001$.

Fig. S5

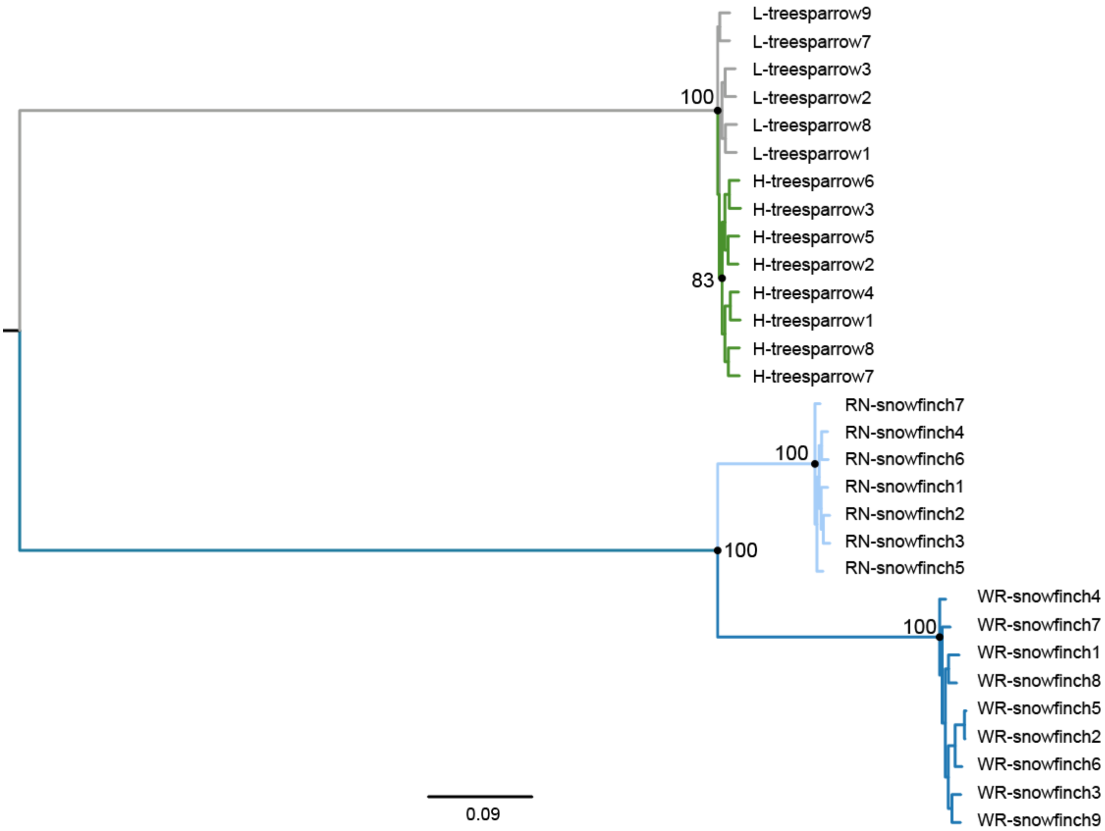

Fig. S5. A phylogenetic tree incorporating all individuals constructed by RaxML. To estimate the phylogenetic relationship, we extracted SNPs from transcriptomic data and run a maximum likelihood phylogenetic tree using the GTR+GAMMA model with 100 bootstraps was run in RAxML v. 8.1. Bootstrap value for each of the high-elevation taxa was shown.

Fig. S6

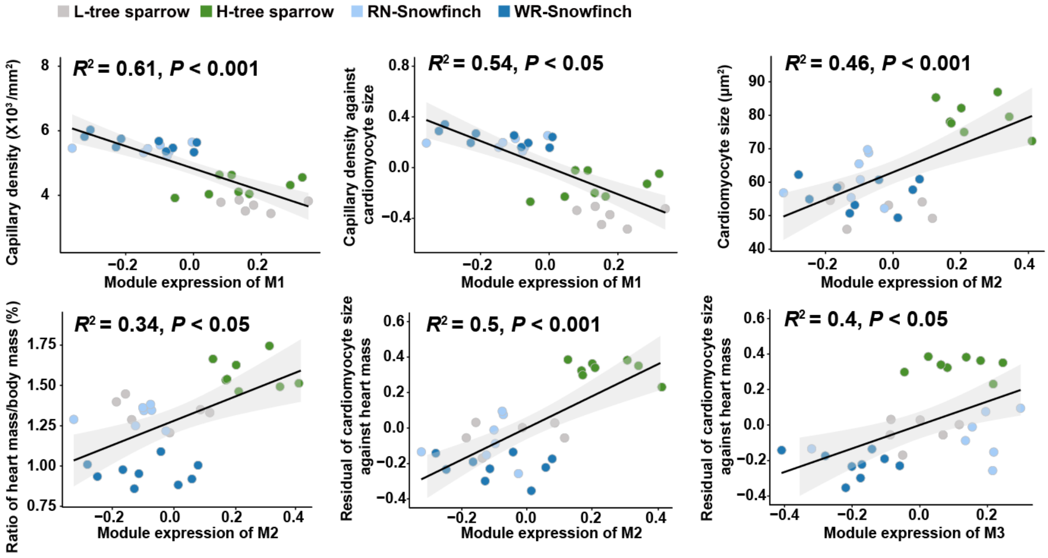

Fig. S6. Relationship of module gene expression and cardiovascular phenotypic change across species.

Fig. S7.

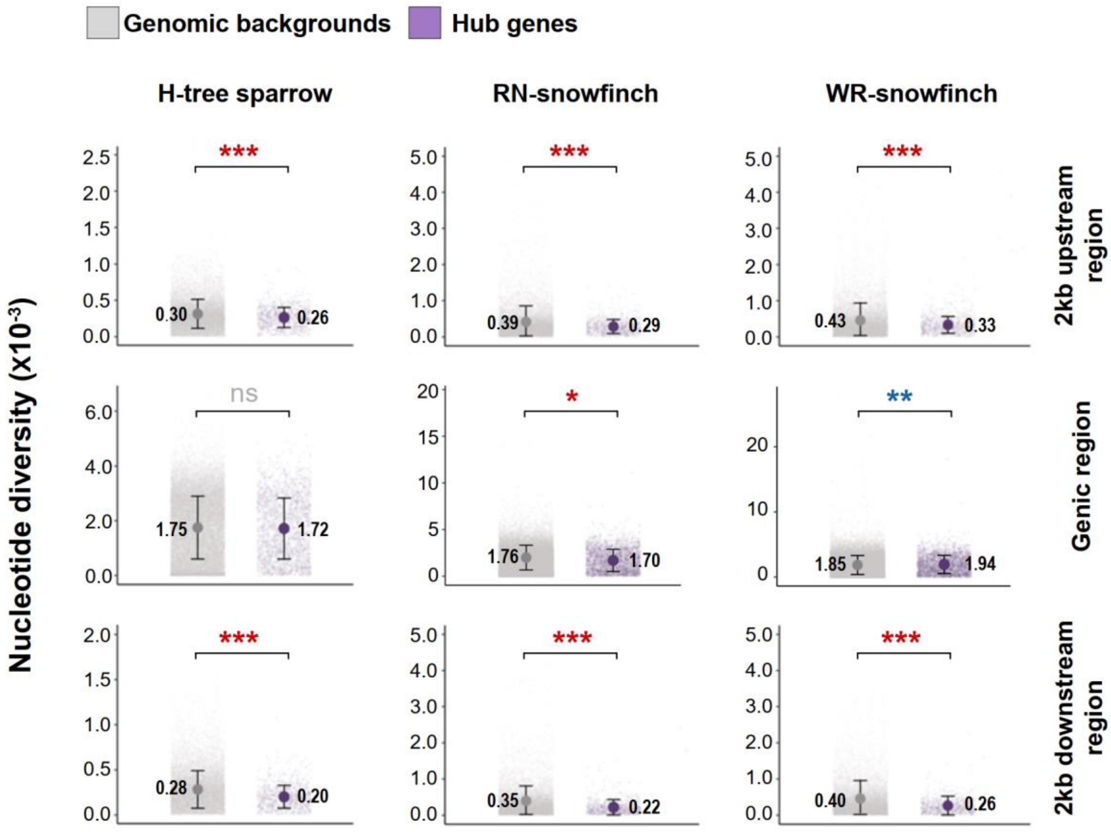

Fig. S7. The regulatory regions of the hub genes exhibited low nucleotide diversity compared to those of the genomic backgrounds in each of the three high-elevation taxa, as indicated by red significant symbols (Wilcoxon Rank-Sum test, ns, non-significant, *, $P < 0.05$; ***, $P < 0.001$). An exception was observed in the genic region of the hub genes in the WR-snowfinch, which showed high nucleotide diversity compared to genomic background, as indicated by a blue statistic symbol ($P < 0.01$). The mean nucleotide diversity values for both the hub genes and genomic backgrounds are given.

Fig. S8

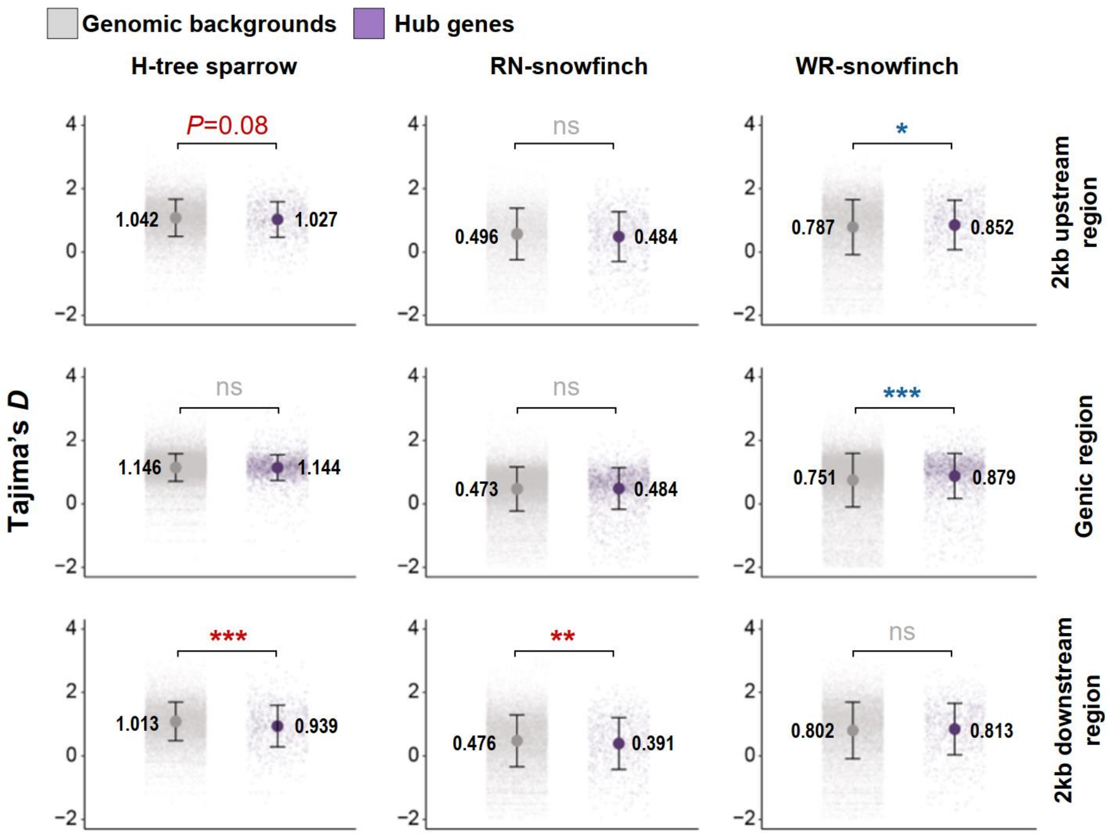

Fig. S8. The 2kb downstream regions of the hub genes exhibited low Tajima's *D* values in H-tree sparrow and RN-snowfinch compared to those of the genomic backgrounds, as indicated by red statistical symbols. In contrast, for the WR-snowfinch, the Tajima's *D* values of the 2kb upstream and genic regions of the hub genes were higher than those of genomic backgrounds, as indicated by blue statistical symbols. The mean Tajima's *D* values for both the hub genes and the genomic backgrounds are provided. Statistical significance was assessed using the Wilcoxon Rank-Sum test (ns, non-significant, *, $P < 0.05$; **, $P < 0.01$; ***, $P < 0.001$).

Fig. S9.

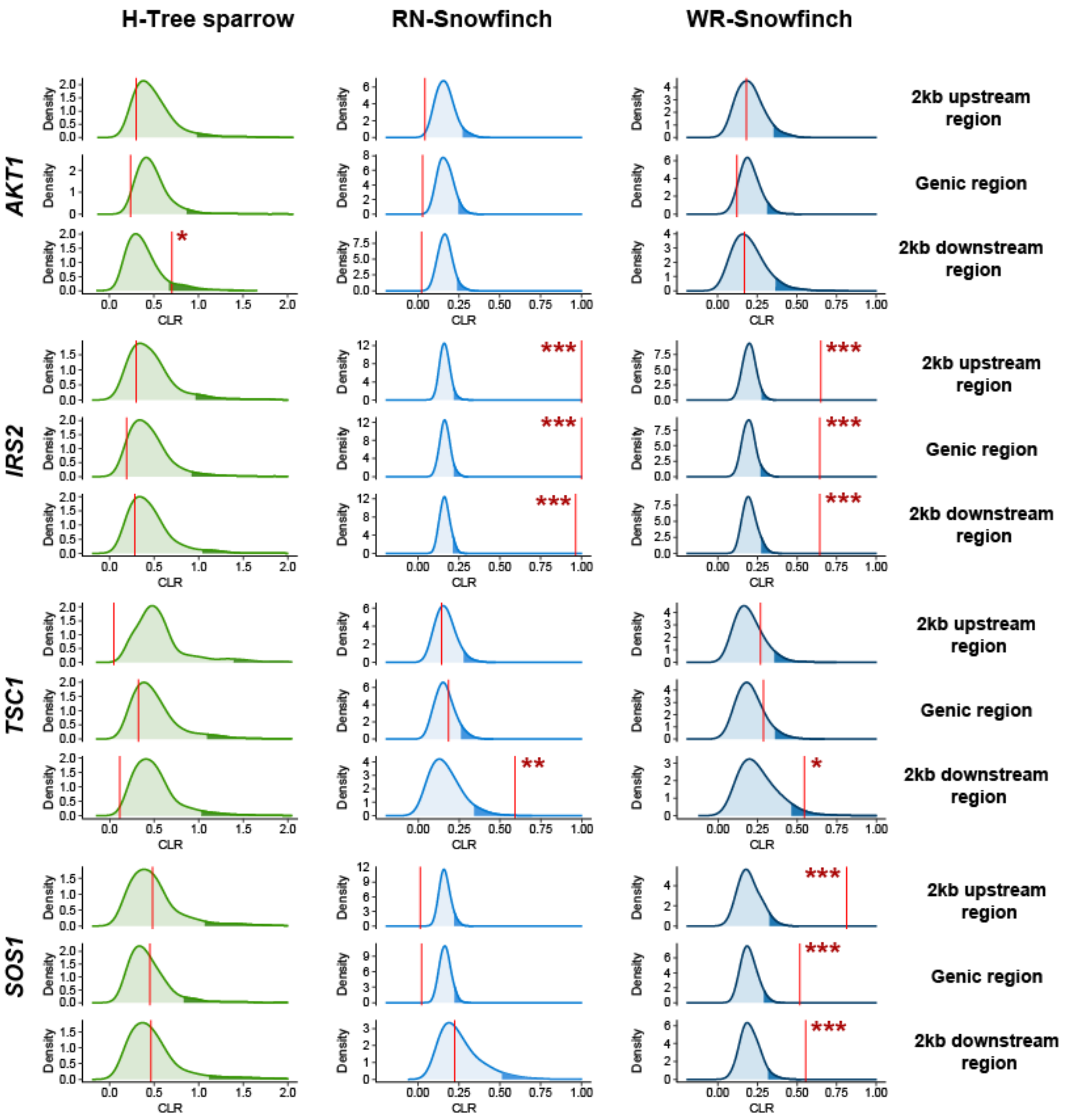
H-Tree sparrow
RN-Snowfinch
WR-Snowfinch
AKT1
IRS2
TSC1
SOS1
2kb upstream region
Genic region
2kb downstream region
Density
CLR
*
**
***

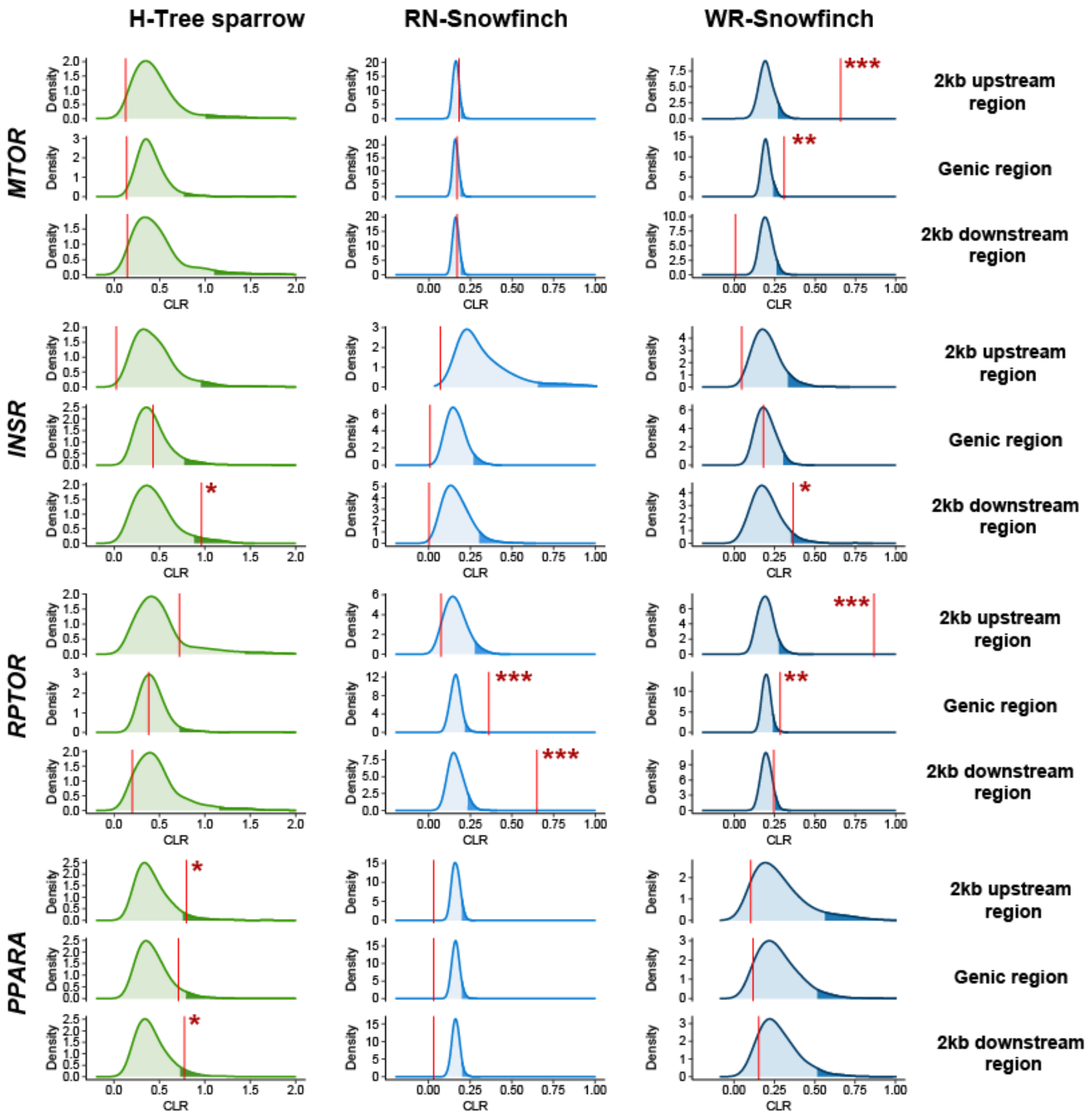

Fig. S9. Eight genes in the diabetic cardiomyopathy and insulin signaling pathways exhibited the signal of selective sweep in their regulatory or genic regions in one or two of the high-elevation taxa. This is indicated by the increased empirical CLR values (vertical red lines) compared to null distributions from 1,000 permutations (*, > 95th percentiles of the null distribution, shown as the dark shaded region of each null distribution; **, > 99th percentile of the null distribution).

Fig. S10

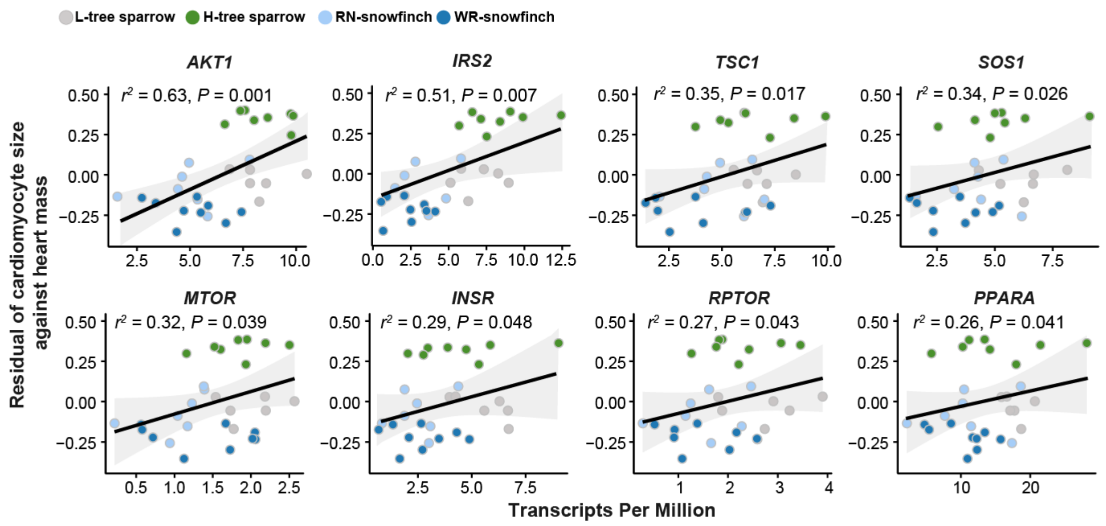

Fig. S10. Eight genes in the diabetic cardiomyopathy and insulin signaling pathways showed the strongest association between expression and heart phenotype (residual of cardiomyocyte size against heart mass).

Fig. S11

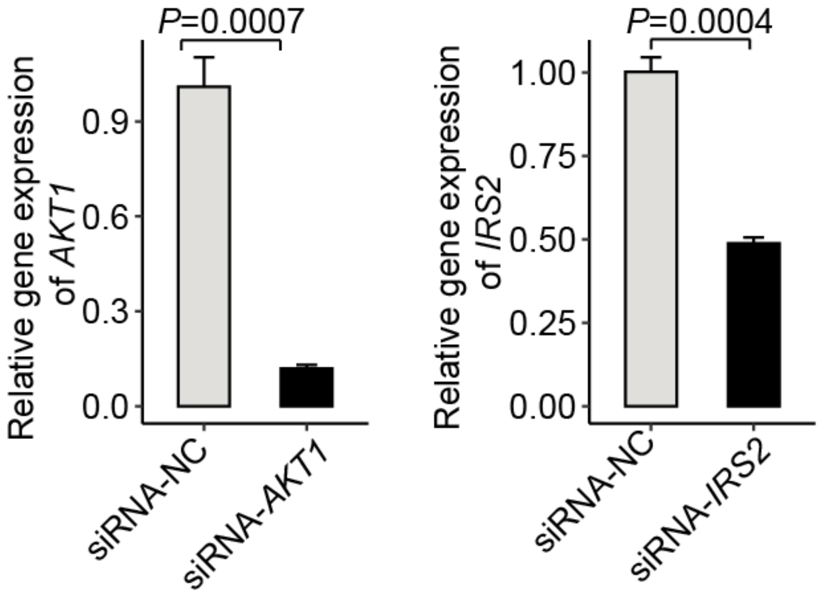

Fig. S11. After transfecting siRNA-*AKT1* and siRNA-*IRS2* and the negative control (siRNA-NC) for 24h, we evaluated knockdown efficiency using quantitative PCR. The mRNA levels of the siRNA-*AKT1* and siRNA-*IRS2* were significantly reduced as compared to the siRNA-NC.

Fig. S12

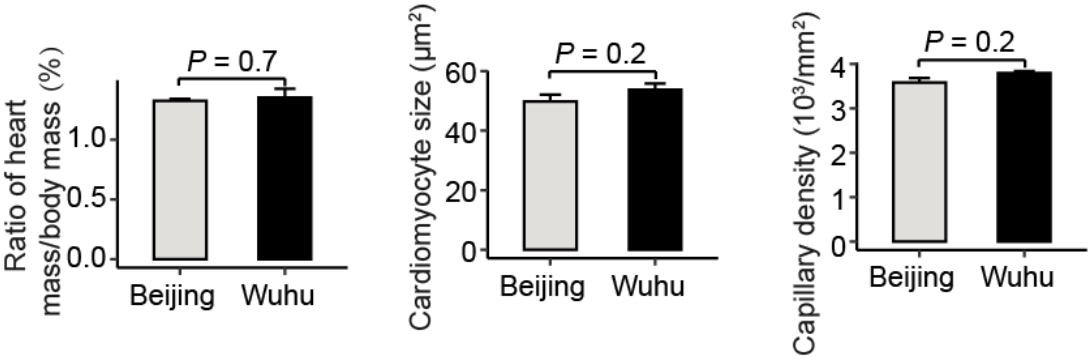

Fig. S12. The ratio of heart mass/body mass, cardiomyocyte size and capillary density did not differ between the individuals from the two low-elevation localities, Beijing and Wuhu. Statistical significance was assessed using Student's *t*-test.

Supplementary Table S1. Sampling information of the WR-snowfinch, RN-snowfinch, high-elevation and low-elevation tree sparrow used in this study.

See appendix Excel file.

Supplementary Table S2. The ratio of heart mass/body mass of WR-snowfinch, RN-snowfinch, low-elevation and high-elevation tree sparrows. Data are presented as means ± standard error (SE).

Morphological measures	Low-elevation tree sparrow	High-elevation tree sparrow	WR-snowfinch	RN-snowfinch
Ratio of heart mass/body mass	1.34 % ± 0.03%	1.57% ± 0.03%	0.96% ± 0.02%	1.31% ± 0.02%

Supplementary Table S3. Cardiomyocyte size (cross-sectional area) and the residual of cardiomyocyte size against heart mass of WR-snowfinch, RN-snowfinch, low-elevation and high-elevation tree sparrows. Data are presented as mean ± standard error (SE).

Morphological measures	Low-elevation tree sparrow	High-elevation tree sparrow	WR-snowfinch	RN-snowfinch
Cardiomyocyte size (μm^2)	52.64 ± 1.84	79.63 ± 1.76	61.34 ± 2.6	56.49 ± 1.55
The residual of cardiomyocyte against heart mass	-0.03 ± 0.03	0.33 ± 0.02	-0.22 ± 0.02	-0.07 ± 0.05

Supplementary Table S4. Capillary density and the residual of capillary density against cardiomyocyte size of WR-snowfinch, RN-snowfinch, low-elevation and high-elevation tree sparrows. Data are presented as means ± standard error (SE).

Morphological measures	Low-elevation tree sparrow	High-elevation tree sparrow	WR-snowfinch	RN-snowfinch
Capillary density (per mm^2)	3684.82 ± 70.44	4280.37 ± 103.83	5429.11 ± 55.00	5619.78 ± 75.81
The residual of capillary density against cardiomyocyte size	-0.38 ± 0.03	-0.14 ± 0.04	0.23 ± 0.02	0.19 ± 0.02

Supplementary Table S5. WGCNA identified three modules of co-expressed genes for which expression changes were significantly associated with cardiovascular phenotypes (ME turquoise, MEgrey60 and ME yellow, indicated by bold; called M1, M2 and M3, respectively, in Figure 2). Regression coefficient and *P* value (in bracket) are given.

Modules	Cardiomyocyte size	Ratio of heart mass/body mass	Residual of cardiomyocyte size against heart mass	Capillary density	Residual of capillary density against cardiomyocyte size
MEpaleturquoise	0.04 (0.31)	0.03 (0.34)	0.1 (0.09)	0.07 (0.17)	0.06 (0.2)
MEdarkgreen	0.08 (0.13)	0.07 (0.17)	0.11 (0.07)	0.06 (0.19)	0.05 (0.23)
MEred	0.02 (0.24)	-0.01 (0.86)	-0.02 (0.78)	0.08 (0.12)	0.09 (0.11)
MEroyalblue	0.08 (0.05)	-0.06 (0.34)	-0.02 (0.17)	0.15 (0.04)	0.17 (0.02)
MEsalmon	0 (0.79)	-0.02 (0.81)	-0.01 (0.88)	-0.04 (0.66)	-0.03 (0.69)
MEcyan	0.03 (0.18)	0 (0.8)	-0.02 (0.15)	0.09 (0.11)	0.1 (0.09)
MEgreen	0.12 (0.05)	0.11 (0.01)	0.04 (0.15)	0.22 (0.01)	0.22 (0.01)
MEpurple	-0.01 (0.58)	0.02 (0.43)	-0.02 (0.91)	0.09 (0.12)	0.09 (0.12)
MEviolet	0.15 (0.03)	0.15 (0.03)	0.14 (0.04)	-0.08 (0.27)	-0.07 (0.23)
MEblack	0.13 (0.02)	-0.05 (0.15)	0.09 (0.01)	0.19 (0.02)	0.22 (0.01)
**MEturquoise**	0.49 (0.54)	0.48 (0.53)	0.48 (0.82)	**0.61 (0.00)**	**0.54 (0.04)**
MEmagenta	0.03 (0.48)	0.13 (0.06)	0.07 (0.15)	0.04 (0.32)	0.03 (0.47)
**MEgrey60**	**0.46 (0.00)**	**0.34 (0.02)**	**0.5 (0.00)**	0.21 (0.04)	0.25 (0.02)
**MEyellow**	0.37 (0.11)	0.38 (0.08)	**0.4 (0.05)**	0.31 (0.67)	0.32 (0.44)
MEsaddlebrown	0.02 (0.55)	0.05 (0.24)	0.07 (0.15)	0.11 (0.07)	0.1 (0.09)
MEwhite	0.09 (0.73)	0.25 (0.01)	0.12 (0.36)	0.12 (0.35)	0.11 (0.43)
MEdarkturquoise	0.03 (0.5)	0.14 (0.04)	0.1 (0.09)	0.09 (0.11)	0.08 (0.17)
MEdarkred	0.1 (0.27)	0.26 (0)	0.14 (0.09)	0.03 (0.71)	0.04 (0.83)
MElightgreen	0.08 (0.79)	0.16 (0.03)	0.09 (0.64)	0.1 (0.47)	0.09 (0.54)
MElightcyan	0.07 (0.4)	0.19 (0.02)	0.1 (0.17)	0.02 (0.81)	0.04 (0.95)
MEblue	0.67 (0.65)	0.68 (0.31)	0.67 (0.74)	0.67 (0.42)	0.67 (0.59)
MEgreenyellow	0.08 (0.41)	0.22 (0.01)	0.09 (0.37)	0.03 (0.48)	0.04 (0.69)
MElightyellow	0.05 (0.85)	0.15 (0.03)	0.05 (0.88)	0.14 (0.11)	0.12 (0.17)
MEpink	0.01 (0.84)	0.1 (0.09)	0.04 (0.33)	0.05 (0.27)	0.04 (0.33)
MEdarkgrey	0.07 (0.15)	0.05 (0.22)	0.06 (0.19)	0 (0.95)	0 (0.94)
MEorange	0 (0.66)	-0.02 (0.71)	-0.01 (0.91)	0.04 (0.29)	0.04 (0.26)
MEtan	0.07 (0.51)	0.04 (0.79)	0.07 (0.47)	0.05 (0.63)	0.05 (0.68)
MEskyblue	0.02 (0.76)	0 (0.9)	0.02 (0.63)	0.02 (0.72)	0.02 (0.7)
MEdarkolivegreen	0.08 (0.39)	0.05 (0.5)	0.08 (0.37)	0.03 (0.87)	0.04 (0.93)
MEmidnightblue	0.12 (0.16)	0.06 (0.36)	0.14 (0.08)	0.04 (0.96)	0.04 (0.89)
MEdarkorange	0.05 (0.63)	0.04 (0.69)	0.05 (0.5)	0.06 (0.43)	0.06 (0.47)
MEbrown	0.82 (0.84)	0.82 (0.72)	0.82 (0.96)	0.82 (0.6)	0.82 (0.72)
MEsteelblue	0.2 (0.51)	0.18 (0.78)	0.2 (0.43)	0.2 (0.5)	0.2 (0.49)
MEgrey	0.04 (0.29)	0.04 (0.32)	0.03 (0.36)	0.01 (0.7)	0.01 (0.69)

Supplementary Table S6. Eight genes in the diabetic cardiomyopathy and insulin signaling pathways are functionally related to cardiac function and growth. Two genes (*AKT1* and *IRS2*) used in functional assay are shown in bold.

Gene symbol	R^2 of LME	Gene-trait Significance	K_{ME}	Function details	Reference
AKT1	**0.63**	**0.40**	**0.93**	**Overexpression of AKT1 can induce cardiac hypertrophy**	**(1)**
IRS2	**0.51**	**0.45**	**0.84**	**Regulation of cardiac size**	**(2)**
TSC1	0.35	0.44	0.89	Smooth muscle protein-22-mediated deletion of Tsc1 results in cardiac hypertrophy	(3)
SOS1	0.34	0.48	0.87	Dilated Cardiomyopathy	(4)
MTOR	0.32	0.28	0.87	Inhibition of mTORC1 activity reduces pathological hypertrophy in response to pressure overload	(5, 10)
INSR	0.29	0.21	0.81	Cardiomyocyte-specific reduction of Insr expression attenuates systolic dysfunction and cardiac growth	(6, 9)
RPTOR	0.27	0.30	0.83	Raptor Ablation Impairs Adaptive Hypertrophy, Alters Metabolic Gene Expression, and Causes Heart Failure in Mice	(7)
PPARA	0.26	0.29	0.84	Cardiac hypertrophy is enhanced in PPAR alpha-/- mice in response to chronic	(8)

pressure overload

1. G. Condorelli *et al.*, Akt induces enhanced myocardial contractility and cell size in vivo in transgenic mice. *Proc. Natl. Acad. Sci. U. S. A.* **99**, 12333-8 (2002).
2. C. Riehle *et al.*, Insulin receptor substrates are essential for the bioenergetic and hypertrophic response of the heart to exercise training. *Mol. Cell. Biol.* **34**, 3450-60 (2014).
3. H. M. Zhang *et al.*, Moderate lifelong overexpression of tuberous sclerosis complex 1 (TSC1) improves health and survival in mice. *Sci. Rep.* **7**, 834 (2017).
4. P. C. Chen *et al.*, Activation of multiple signaling pathways causes developmental defects in mice with a Noonan syndrome–associated Sos1 mutation. *J. Clin. Invest.* **120**, 4353-4365 (2010).
5. S. Sciarretta *et al.*, New Insights Into the Role of mTOR Signaling in the Cardiovascular System. *Circ. Res.* **122**, 489-505 (2018).
6. D. M. S. Díaz *et al.*, The Insulin-like Growth Factor Signalling Pathway in Cardiac Development and Regeneration. *Int. J. Mol. Sci.* **23**, 234 (2021).
7. P. Shende *et al.*, Cardiac Raptor Ablation Impairs Adaptive Hypertrophy, Alters Metabolic Gene Expression, and Causes Heart Failure in Mice. *Circulation* **123**, 1073-1082 (2011).
8. P. J. H. Smeets *et al.*, Cardiac hypertrophy is enhanced in PPAR -/- mice in response to chronic pressure overload. *Cardiovasc. Res.* **78**, 79-89 (2008).
9. I Shimizu *et al.*, Excessive cardiac insulin signaling exacerbates systolic dysfunction induced by pressure overload in rodents. *J. Clin. Invest.* **120**, 1506-1514 (2010).
10. W. H. Shen *et al.*, Cardiac Restricted Overexpression of Kinase-dead Mammalian Target of Rapamycin (mTOR) Mutant Impairs the mTOR-mediated Signaling and Cardiac Function. *J. Biol. Chem.* **283**, 13842-13849 (2008).

Supplementary Table S7. siRNA sequences used in the functional assay.

**Primer ID**	**Sequence (5' to 3')**
Gallus gallus_AKT1	GCUCUUUGAACUCAUCCUUAU
Gallus gallus_IRS2	GCGAUGAUUACAUGCCCAUGA
*Gallus gallus*_Negative control	ACGUGACACGUUCGGAGAATT

Supplementary Table S8. The primer sequences used in RT-PCR.

**Primer ID**	**Sequence(5'to3')**
Passer montanus_GAPDH-F	GGTGCTGCTCAGAACATTATCC
Passer montanus_GAPDH-R	CATCGTACTTGGCTGGCTTTT
Pyrgilauda ruficollis_GAPDH-F	TGGAGGGATGGCAGAGGT
Pyrgilauda ruficollis_GAPDH-R	TTCCCACAGCCTTAGCAG
Onychostruthus taczanowskii_GAPDH-F	TGGAGGGATGGCAGAGGT
Onychostruthus taczanowskii_GAPDH-R	CAGACGGCAAGTCAGGTC
Passer montanus_IRS2-F	AACCAGGGCGTCAGGAAGT
Passer montanus_IRS2-R	CAGGAGTCCAGGGCAATCAC
Pyrgilauda ruficollis_IRS2-F	GGTTCGTGCGCCTCAATT
Pyrgilauda ruficollis_IRS2-R	GGATGGTCTCGTGGATGTTCT
Onychostruthus taczanowskii_IRS2-F	TACCTCATCGCCCTCTACACCA
Onychostruthus taczanowskii_IRS2-R	CCTCCTGCTCCTGCTCGTT
Passer montanus_AKT1-F	AACTGGACGGTATTACGC
Passer montanus_AKT1-R	AGCTGTTAGGAACGGGTG
Pyrgilauda ruficollis_AKT1-F	AACTGGACGGTATTACGC
Pyrgilauda ruficollis_AKT1-R	AGCTGTTAGGAATGGGTG
Onychostruthus taczanowskii_AKT1-F	GTTCTGGAGGATAATGACT
Onychostruthus taczanowskii_AKT1-R	CCTGATTGTAGAAAGGGAG
Gallus gallus_ANP-F	AGACTGGAGGATAAGTTTGC
Gallus gallus_ANP-R	CTCATCGCTGTCATCTGTG
Gallus gallus_MYH7-F	CAAGTCGGCTTACCTGATG
Gallus gallus_MYH7-R	ACCTGCTGGACGCTCTGT
Gallus gallus_IRS2-F	GCCCAGAACATCCACGAGAC
Gallus gallus_IRS2-R	AGGAAGAGGAGGACGACGACT
Gallus gallus_AKT1-F	ATAATGACTATGGTCGTGC
Gallus gallus_AKT1-R	AAAGTGCGTGGAAATCTA
Gallus gallus_GAPDH-F	GCCCAGAACATCATCCCA
Gallus gallus_GAPDH-R	CGGCAGGTCAGGTCAACA
